# Differential Effects of Toll-Like Receptor Activation and Differential Mediation by MAP Kinases of Immune Responses in Microglial Cells

**DOI:** 10.1007/s10571-021-01127-x

**Published:** 2021-07-23

**Authors:** Jaedeok Kwon, Christos Arsenis, Maria Suessmilch, Alison McColl, Jonathan Cavanagh, Brian J. Morris

**Affiliations:** 1 Institute of Neuroscience and Psychology, College of Medical, Veterinary and Life Sciences, University of Glasgow, West Medical Building, Glasgow, G12 8QQ UK; 2 Institute of Infection, Immunity and Inflammation, University of Glasgow, Glasgow, UK

**Keywords:** Microglia, Viral infection, Bacterial infection, Cytokines, Chemokines, Signaling Pathway, Poly (IC)

## Abstract

Microglial activation is believed to play a role in many psychiatric and neurodegenerative diseases. Based largely on evidence from other cell types, it is widely thought that MAP kinase (ERK, JNK and p38) signalling pathways contribute strongly to microglial activation following immune stimuli acting on toll-like receptor (TLR) 3 or TLR4. We report here that exposure of SimA9 mouse microglial cell line to immune mimetics stimulating TLR4 (lipopolysaccharide—LPS) or TLR7/8 (resiquimod/R848), results in marked MAP kinase activation, followed by induction of nitric oxide synthase, and various cytokines/chemokines. However, in contrast to TLR4 or TLR7/8 stimulation, very few effects of TLR3 stimulation by poly-inosine/cytidine (polyI:C) were detected. Induction of chemokines/cytokines at the mRNA level by LPS and resiquimod were, in general, only marginally affected by MAP kinase inhibition, and expression of *TNF*, *Ccl2* and *Ccl5* mRNAs, along with nitrite production, were enhanced by p38 inhibition in a stimulus-specific manner. Selective JNK inhibition enhanced Ccl2 and Ccl5 release. Many distinct responses to stimulation of TLR4 and TLR7 were observed, with JNK mediating TNF protein induction by the latter but not the former, and suppressing Ccl5 release by the former but not the latter. These data reveal complex modulation by MAP kinases of microglial responses to immune challenge, including a dampening of some responses. They demonstrate that abnormal levels of JNK or p38 signalling in microglial cells will perturb their profile of cytokine and chemokine release, potentially contributing to abnormal inflammatory patterns in CNS disease states.

**Supplementary Information:**

The online version contains supplementary material available at 10.1007/s10571-021-01127-x.

## Introduction

Inappropriate microglial activation is a component of most neurodegenerative diseases. In Alzheimer’s disease, for example, activated microglia surround amyloid plaques, and genetic evidence implies strongly that microglia directly influence the neurodegenerative process (Yeh et al. 2017; Efthymiou and Goate 2017; Baufeld et al. 2018). In certain psychiatric diseases, most prominently schizophrenia, there is also evidence that microglia contribute to disease aetiology (Takahashi et al. 2016; Herron et al. 2018). The epidemiological evidence that exposure to viral infection in utero increases schizophrenia risk in offspring is consistent with inappropriate microglial activation during development as a component of disease causation.

In Alzheimer’s disease (AD), there are many reports of abnormal levels of chemokines/cytokines in plasma, cerebrospinal fluid and post-mortem tissue (Swardfager et al. 2010), although many are contradictory. Conversely, equivalent studies in schizophrenia consistently indicate elevated CNS cytokine and chemokine expression (Zhang et al. 2002; Miller et al. 2011; Hong et al. 2017). For example, robust increases in interleukin 6 (IL-6) are detected in cerebrospinal fluid in patients samples, whilst in post-mortem tissue, increased inflammatory markers such as *CXCL8*, *IL-1β*, and *IL-6* mRNAs are detected in dorsolateral prefrontal cortex from patients (Fillman et al. 2013).

The possibility of microglial involvement in a range of CNS diseases becomes of significant interest considering that complementary evidence links signalling via the JNK subgroup of MAP kinase cascades to the aetiology of the same diseases. JNK phosphorylation (activation) is elevated in neurons showing early signs of Aβ1-42 accumulation in AD and is also increased in prefrontal cortex and hippocampus in later stage AD (Del Villar and Miller 2004; Swatton et al. 2004; Mufson et al. 2012), and JNKs are considered as potential therapeutic targets (Yarza et al. 2016). JNK activation is also heavily implicated in the neurotoxic responses to CNS-penetrant viruses, such as polio virus, west nile virus, Human immunodeficiency virus (HIV) and Herpes simple virus (HSV) (Michaelis et al. 2007; Autret et al. 2008; Crews et al. 2009; Cliffe et al. 2015; Beckham et al. 2007). Genetic evidence supports the involvement of JNK signalling in schizophrenia aetiology (Winchester et al. 2012; Morris and Pratt 2014).

Toll-like receptors (TLRs), which detect the presence of tissue pathogens such as bacteria and viruses, are expressed by immune cells including monocytes and macrophages, and also in CNS microglia. Many signalling pathways interact to regulate microglial activation in response to immune challenge. Most prominent amongst these are the nuclear factor kappa-light-chain-enhancer of activated B cells (NF-κB) and MAP kinase (ERK, p38 and JNK) cascades, which combine to induce proliferation, morphological change, migration, and synthesis and release of inflammatory mediators including nitric oxide, cytokines and chemokines (Dong et al. 2002). The JNK pathway is known to contribute to the activation of T cells and macrophages, but the extent to which JNK signalling is involved in microglial activation is less clear. For example, induction of TNF, IL-2, and CCL5 following immune challenge is reduced in mice lacking JNK1 (Tran et al. 2006). Conversely, induction of inducible nitric oxide synthase (NOS2, iNOS) and IL-6 is enhanced in bone-marrow-derived macrophages (BMDMs) from mice lacking JNK1 (Zhao et al. 2017).

Most studies compare the effects of TLR4, responding to bacterial mimetics such as lipopolysaccharide (LPS) and TLR3, responding to double-stranded virus mimetics such as poly inosine-cytosine (poly I:C). There is limited information concerning responses to mimetics of single-stranded viruses, such as resiquimod/R848 (Hemmi et al. 2002), which act on TLR7 and TLR8. In view of epidemiological evidence linking single-stranded virus exposure to psychiatric and neurological diseases, this is an important area that needs to be addressed.

In pursuit of an improved understanding of the mechanisms involved in microglial activation, we were also keen to assess the extent to which MAP kinase signalling, and in particular the JNK pathway, mediates microglial responses to immune challenge. There is a concern that viral immortalisation causes established microglial cell lines to be unrepresentative of their in vivo counterparts (Henn et al. 2009; Stansley et al. 2012; Butovsky et al. 2014; Das et al. 2016). Most of these lines stem from primary microglia cultures derived from the brain or the spinal cord, which were immortalized by viral transduction with oncogenes, e.g. BV2 (Blasi et al. 1990) or N9 (Righi et al. 1989) cell lines. This is a particular issue for studying MAP kinase involvement in cell responses, since in many cases the immortalisation involves increasing MAP kinase activity. Whilst advantages of cell lines include their ease of maintenance and their abundant availability due to their unrestricted proliferative capacity, a major disadvantage is that viral transformation or immortalization may alter the microglial phenotype. Recent studies have pointed out that microglia cell lines differ both genetically and functionally from primary microglia and ex vivo microglia (Butovsky et al. 2014; Das et al. 2016; Melief et al. 2016). The SIM-A9 cell line derived from a population of spontaneously transformed microglia in a primary mouse cortical culture (Nagamoto-Combs et al. 2014), and hence may be more closely related to microglia in vivo. In this study, we use SIM-A9 mouse microglial cells to probe the similarities and differences between activation of TLR3, TLR4 and TLR7/8, and the contribution of MAP kinase pathways to microglial responses.

## Materials and Methods

### Cell Culture and Stimulation

Murine microglial cells (SIM-A9) (RRID:CVCL_5I31) (Nagamoto-Combs et al. 2014) were purchased from ATCC (CRL3265). To expand the population, the cells were grown in DMEM:F12 (Gibco 302006) containing 5% horse serum (Gibco-26050-088) with 1% penicillin/streptomycin. The cells were detached with Trypsin/EDTA. For the immune stimulation describe below, required drugs were added after horse serum was removed (i.e., serum-free conditions).

LPS (Sigma, L-8274, 50 ng/ml (100 ng/ml for Griess assay), dH_2_O), Poly I:C (LMW, Invivogen, Tlrl-picw, 100 ng/ml, dH_2_O), and resiquimod (Tocris, resiquimod, 3 μM, DMSO) were used to induce immune responses. JNK-IN-8 (Sigma, SML1246, 1 μM, DMSO), PD98059 (Calbiochem, 513000, 40 μM, DMSO), SB203580 (Calbiochem, 559389, 5 μM, DMSO) were used to inhibit specific MAP kinases, and they were added 3 h (JNK-IN-8)(Zhang et al. 2012) or 30 min (PD98059, SB203580) prior to immune stimuli.

### RNA Extraction and RT-qPCR

Total RNA was extracted from the cell culture after various experimental conditions using an RNeasy mini kit (Qiagen, 79254) with additional DNase I (Qiagen, 79254) as per manufacture’s instruction. The RNA concentration was determined by a Nanodrop DeNovix DS-11+ Spectrophotometer. RNA was reverse-transcribed to cDNA using High-capacity RNA-to-cDNA™ kit (Applied Biosystems, 4387406) according to the manufacturer’s instruction.

Gene expression in the cells was quantified Fast SybrGreen™ master mix (Applied Biosystems, 4385612) for each target using the QuantaStudio7 (Thermo Fisher Scientist). Primer sequences are provided in Supplementary Table 1. The level of the target genes was normalised to a geometric mean of two reference genes (*Gapdh, Henmt1*), which were not significantly different within treatment groups, and relative differences in target gene expression were determined using absolute quantification method.

Only for *Tlr* expression analysis, the relative quantification method (2^−ΔΔCT^) was used. The arbitrary value relative to *Gapdh* was compared to the average of all control (no RT) values as an index of relative expression.

### Griess Assay

Cell culture media were collected and transferred to a 96 well plate. The culture media were mixed with an equal volume of the Griess assay reagent (Enzo Life Sciences, ALX-400-004-L050) and incubated for 10–15 min under dark condition. Measurements were taken (Multiskan® Spectrum; Thermo Scientific™, 540 nm), operated via the SkanIt™ software (Thermo Scientific™). 650 nm absorbance was taken for the reference.

### Western Blot

Western blotting was performed essentially as described previously (Guilding et al. 2007). The cells were stimulated with pathogen mimetics for 15 min and proteins were extracted from the stimulated cells using ice-cold RIPA buffer (10 mM Tris–HCl (pH 7.4) + 150 mM NaCl + 1 mM EDTA + 1%(v/v) Triton x-100 + 0.1%(w/v) SDS + 0.5% (w/v) sodium deoxycholate + dH_2_O + 1% (v/v) proteinase inhibitors (Sigma, P8340) + 1 mM Sodium Orthovanadate + 2.5 mM Sodium Pyrophosphate + 25 mM Sodium Fluoride) and the lysate was centrifuged at 8000 × *g* for 10 min at 4 °C. The supernatant was kept at − 20 °C till further use. Proteins were mixed with sample buffer (NuPAGE®, Invitrogen, NP0007) and the reducing agent (NuPAGE®, Invitrogen, 1769410) and heated for 10 min at 80 °C. Preheated samples and protein ladder (Bio-Rad, 161-0375) were loaded onto a pre-cast gel (10% Bis–Tris gel, NuPAGE®, Invitrogen) and transferred to pre-methanol/diluted NuPAGE transfer buffer soaked PVDF membrane (0.45 μm pore size, Novex, LC2005) NUPAGE transfer PVDF membranes. The transferred membranes were blocked by 5% milk/TBS-T for 30 min at room temperature and incubated with primary antibody overnight at 4 °C with gentle shaking (pJNK, Abcam, ab76572, 1:10000; pERK, Cell Signaling Technology (CST), 4377, 1:5000; pp38, CST, 4511, 1:4000; GAPDH Genetex, GTX627408, 1:20000). Specificity of the antibodies was assessed by the presence of a single band of the predicted size (in Kda) in our western blots (Supplementary Figure 5), and by inspection of literature data of immunoreactivity absence in mice with the corresponding gene deleted. The membranes were washed three times for 10 min each in TBS-T and incubated with secondary antibody (anti-Rabbit, Merck 12-348, 1:10000 in 1% milk/TBS-T) for 2 h at room temperature. Washes were repeated after secondary antibody incubation, washing three times for 10 min, first washing in TBS-T and then last two washing in 10 × TBS. Blots were developed via ECL (Millipore, WBKLS0100; or CST, 6883S), and images captured (Syngene PXi).

### Gel Electrophoresis

cDNA samples were mixed with 5 µl of SybrGreen (Applied Biosystems, Fast SYBR™ green master mix, 4385612), 0.3 µl of a target gene of forward primer, 0.3 µl of a target gene of reverse primer, and 3.4 µl of RNase free water. The PCR was 95 °C for 3 min followed by 40 cycles of 95 °C for 3 s, 60 °C for 30 s, and 95 °C for 1 min. The final PCR products were run on a 1.5% agarose gel (120 V, 45 min). The gel was imaged under UV lamp machine (Alpha Innotech, AlphaImage™).

### ELISA

Secreted cytokines and chemokines from the stimulated cells were measured by ELISA kits (IL-6, eBiosciece™, 12364003; TNF-a, eBiosciece™, 155501117; CCL2, eBiosciece™, 15561137; CCL5, R&D system, DY478-05, CXCL10, R&D system, DY466-05) per manufacturers’ instructions. The culture media were stored at − 80 °C if they were not immediately used.

### Statistics

A priori sample size calculations were performed, based on our previous experience with these techniques, assuming a minimum power of 0.8 and significance level of *p* < 0.05 for ANOVA main effects (Minitab). All results are reported as groups means ± SEM. No outliers were removed. Any deviation from normality of distribution (Kolmogorov – Smirnov test) and homogeneity of variance was assessed using Minitab. Non-normally distributed data were log transformed, and two-way or three-way analysis of variance (ANOVA) with post hoc Tukey multiple tests conducted (Minitab). For small sub-group sizes, individual planned comparisons between groups were also performed using a Fisher-Pitman permutation test. Details of the results of the statistical analysis are given in figure legends and in Supplementary Table 2.

## Results

### Resting Microglial Cells Show Low Expression of TLR3 and TLR8

**Fig. 1:**
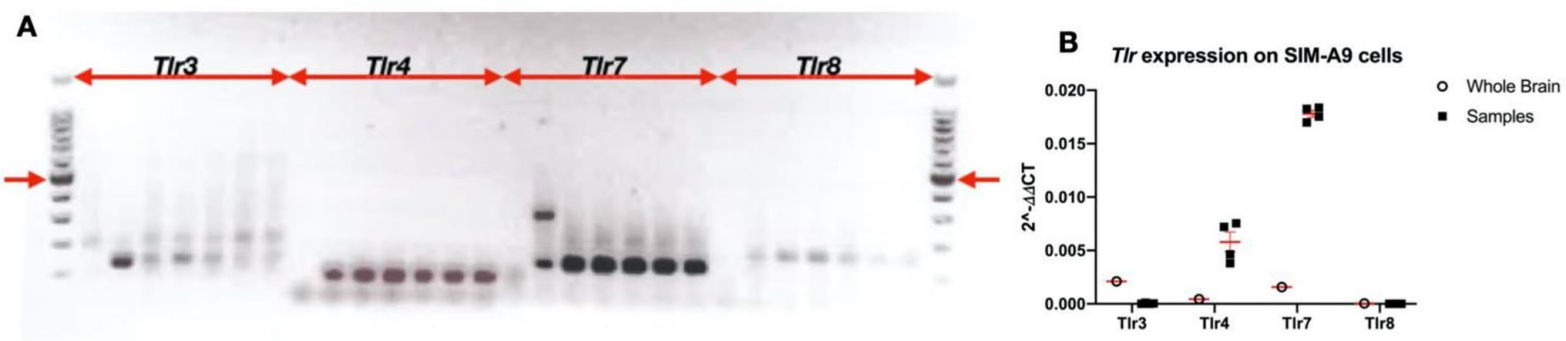
*Tlr* expression in SIM-A9 microglial cells. The cells were maintained under serum-free conditions overnight prior to RNA extraction and PCR. **A** A 100 bp DNA ladder is shown at left and right (red arrow indicates 500 bp). Loading order, negative control, positive control (cDNA from whole mouse brain), and five independent SimA9 cell samples; loading order was repeated for each TLR. **B** Relative quantification of *Tlr* expression on SIM-A9 cell samples, whole brain cDNA was used as a positive control (*n* = 5–6/group)

Previous studies (Bsibsi et al. 2002; Olson and Miller 2004; Trudler et al. 2010) have compared the expression of TLRs in microglial cells maintained in serum, and hence likely to be under some degree of stimulation. To assess the relative expression of TLR3, TLR4, TLR7 and TLR8 in resting microglial cells, grown in serum-free conditions, prior to using agents stimulating these receptors to study the microglial responses, we used PCR to amplify the corresponding mRNAs (Fig. 1). At the mRNA level, *Tlr3* and *Tlr8* mRNAs showed a relatively low expression level compared to *Tlr4* and *Tlr7* mRNAs.

### TLR Stimulation Activates MAPK Pathways

**Fig. 2:**
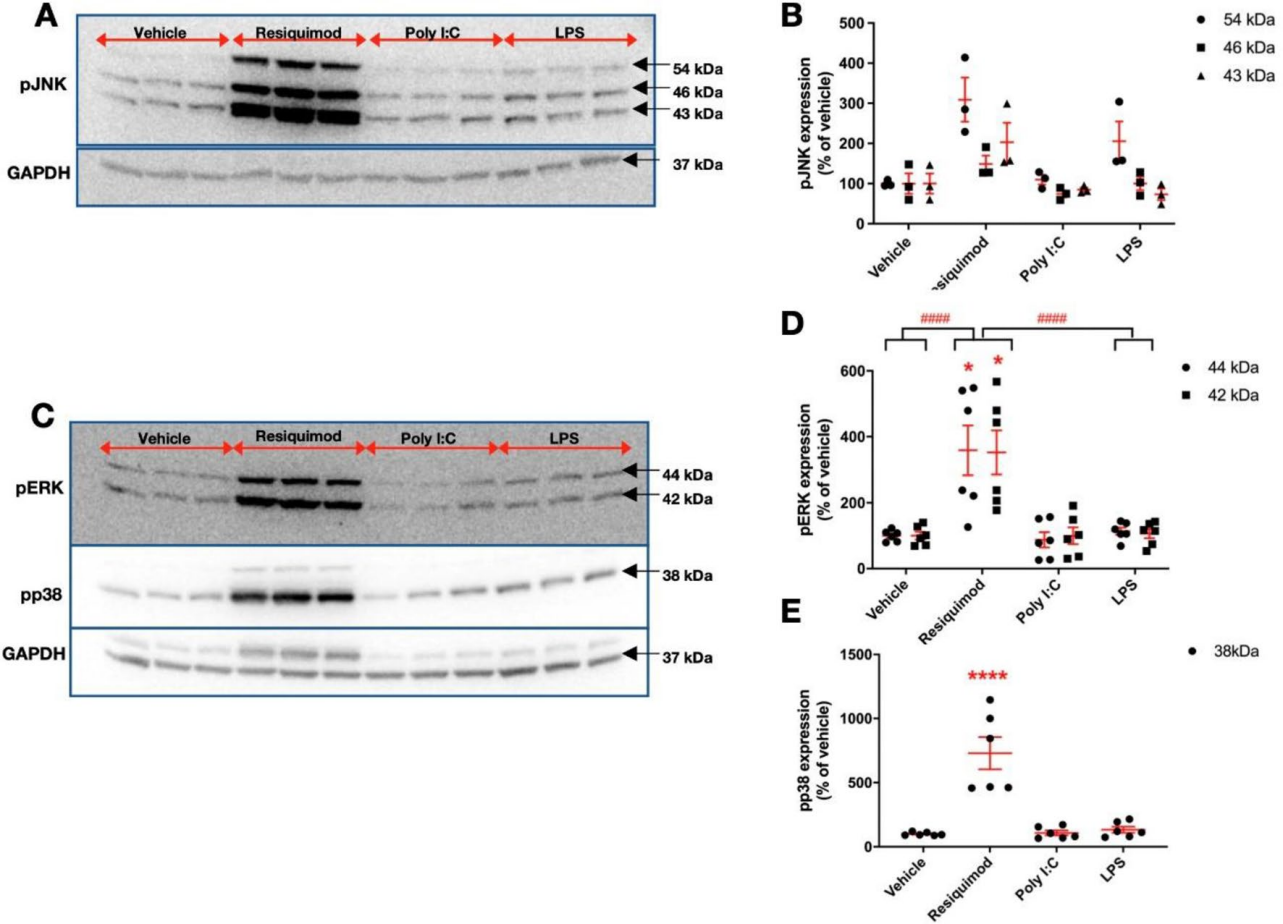
Resiquimod induces MAPK phosphorylation in microglial cells. The microglial cells were cultured in serum-free medium overnight, and treated with LPS (50 ng/ml), poly I:C (100 ng/ml), or resiquimod (3 μM) for 15 min. **A** Resiquimod increases the level of pJNK compared to poly I:C or LPS. **B** The pJNK levels appear increased with resiquimod compared to vehicle. **C** and **D** Resiquimod, but not poly I:C or LPS, increases pERK and pp38 levels significantly. **C** and **E** Resiquimod increased pp38 levels significantly. One representative blot out of two independent experiment images is shown for A and C. Individual dots are shown along with mean ± SEM. The data were analysed by two-way ANOVA, Tukey comparison (pJNK, pERK) and by one-way ANOVA, Bonferroni comparison (pp38) (*p < 0.05, ****p ≤ 0.0001 vs. same isoform in vehicle group; ^####^*p* ≤ 0.0001 overall effect Tukey comparisons). (*n* = 3–6/group). Details of ANOVA *F* values and *p* values and of Fisher-Pitman permutation testing are provided in Supplementary Table 2

We assessed the extent to which immune mimetics were able to activate MAP kinase signalling in the microglial cells. The cells were stimulated with one of three different pathogen mimetics: resiquimod (TLR7/8 agonist) activated pMAPKs 15 min after stimulation (JNK, ERK and p38) (Fig. 2), however LPS and poly I:C did not show any meaningful changes. With a slightly longer exposure time, after 30 min, LPS appeared to produce activation of pMAPKs, poly I:C rarely showed significant changes compared to LPS and resiquimod, even though pp38 appeared to be decreased at 30 min point (Supplementary Figures 1 and 2). The lack of MAPK phosphorylation after poly I:C stimulation may be a result of the low expression of *Tlr3*. These results indicate that microglial TLR4 and TLR7 use MAPKs as part of their response to immune stimuli. MAPKs have different isoforms. These isoforms have distinct expression patterns and their different biological function have been reported (Pagès et al. 1999; Kuan et al. 1999; Coffey 2014). For example, *Jnk1* and *Jnk2* are expressed through the body, although *Jnk3* is expressed in limited organs such as the brain, and heart (Davis 2000). Therefore, it is important to know how individual isoform expression varies. Our results showed that JNK isoforms were significantly changed (Fig. 2A,B), and also both ERK isoforms (Fig. 2C,D).

### Nitric Oxide Production After Immune Challenge

**Fig. 3:**
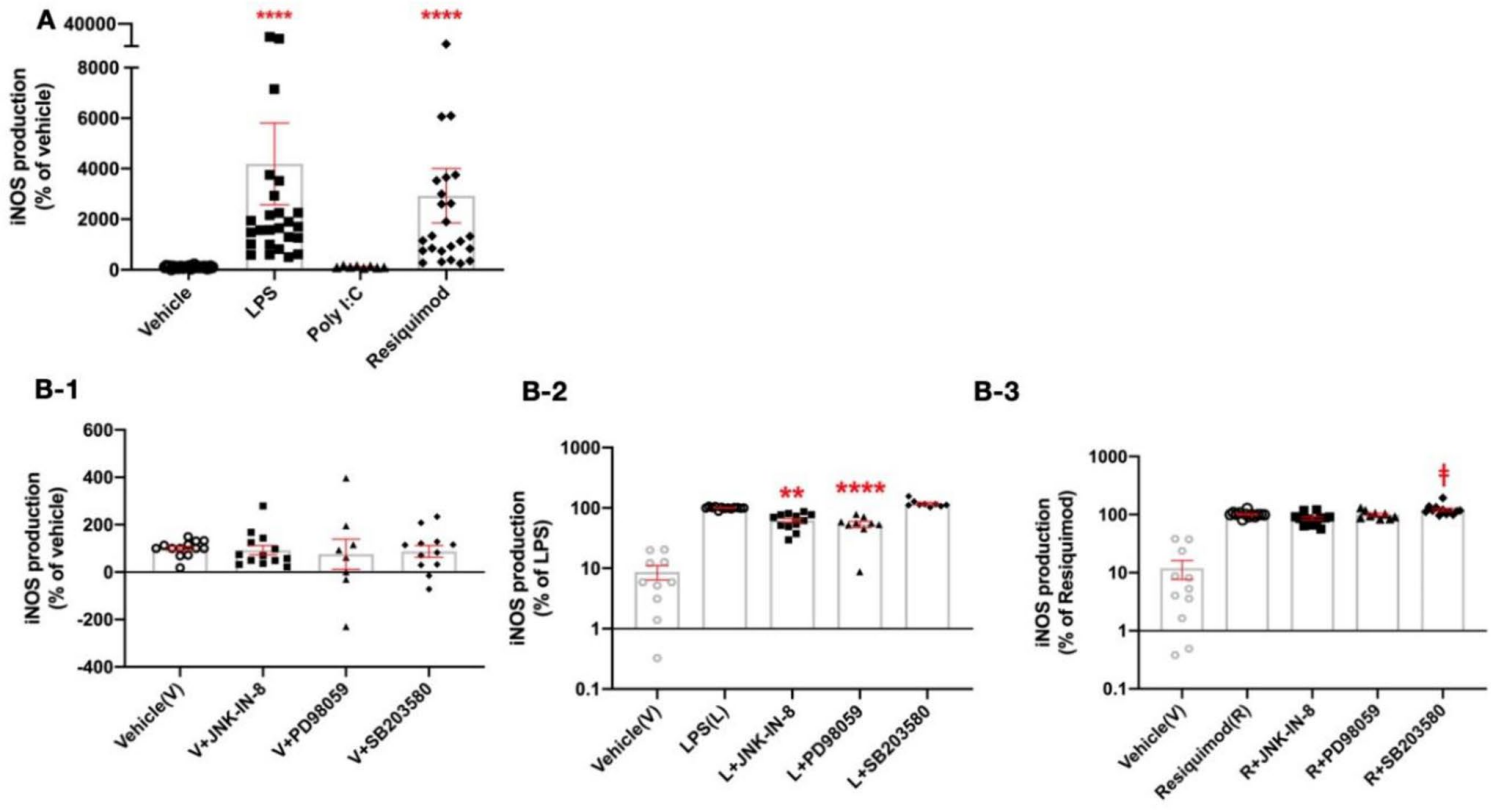
iNOS production. The microglial cells were cultured in serum-free medium and left overnight before use. The cells were stimulated by LPS (100 ng/ml), poly I:C (100 ng/ml) or resiquimod (3 μM) for 24 h and MAPK inhibitors, JNK-IN-8 (1 μM), PD98059 (40 μM), or SB203580 (5 μM), were added before the mimetics. **A** iNOS was increased by LPS and resiquimod, however poly I:C did not produce any affect (**B-1**). Nitrite release in the vehicle condition was not affected by MAPK inhibition (**B-2**), but JNK-IN-8 and ERK inhibition under the LPS condition reduced nitrite release, whilst (**B-3**) p38 inhibition with resiquimod increased iNOS production. Griess assay was performed and the individual data points are shown along with mean ± SEM. The data were analysed by two-way ANOVA, Tukey post hoc test (*p ≤ 0.05, **p ≤ 0.005, ***p ≤ 0.001, ****p ≤ 0.0001 vs. same TLR activator without inhibitors, Tukey comparisons; ^ǂ^*p* ≤ 0.05, ^ǂǂ^*p* ≤ 0.005, ^ǂǂǂ^*p* ≤ 0.001, ^ǂǂǂǂ^*p* ≤ 0.0001 Resiquimod vs. SB203580 within resiquimod treatment group one-way ANOVA Bonferroni post hoc test). Vehicle(V) data in **B-2** and **B-3** are repeated data from Vehicle(V) in **B-1**. (*n* = 8–24/group). Details of ANOVA *F* values and *p* values are provided in Supplementary Information

We then assessed the extent to which immune mimetics were able to stimulate an immune response in the microglial cells To monitor microglial activation after the stimuli, nitrite production was measured as an index of inducible nitric oxide synthase (iNOS) induction. Nitrite production was significantly increased by LPS and resiquimod but not by poly I:C (Fig. 3A). In order to understand MAPK effects on iNOS production, three different inhibitors were given before LPS and resiquimod. JNK and ERK inhibition reduced nitrite production under the LPS condition, but not with resiquimod (Fig. 3B-2, B-3). p38 inhibition tended to increase nitrite production with resiquimod (F(3,42) = 8.175, *p* = 0.0002; resiquimod vs. resiquimod + SB203580, *p* = 0.0439; Bonferroni post hoc) (Fig. 3B-3). This observation suggests differences in MAPK involvement downstream of TLR4 and TLR7/8, with greater involvement of ERK and JNK in TLR4 signalling.

### Time Course of Cytokine and Chemokine Responses

**Fig. 4:**
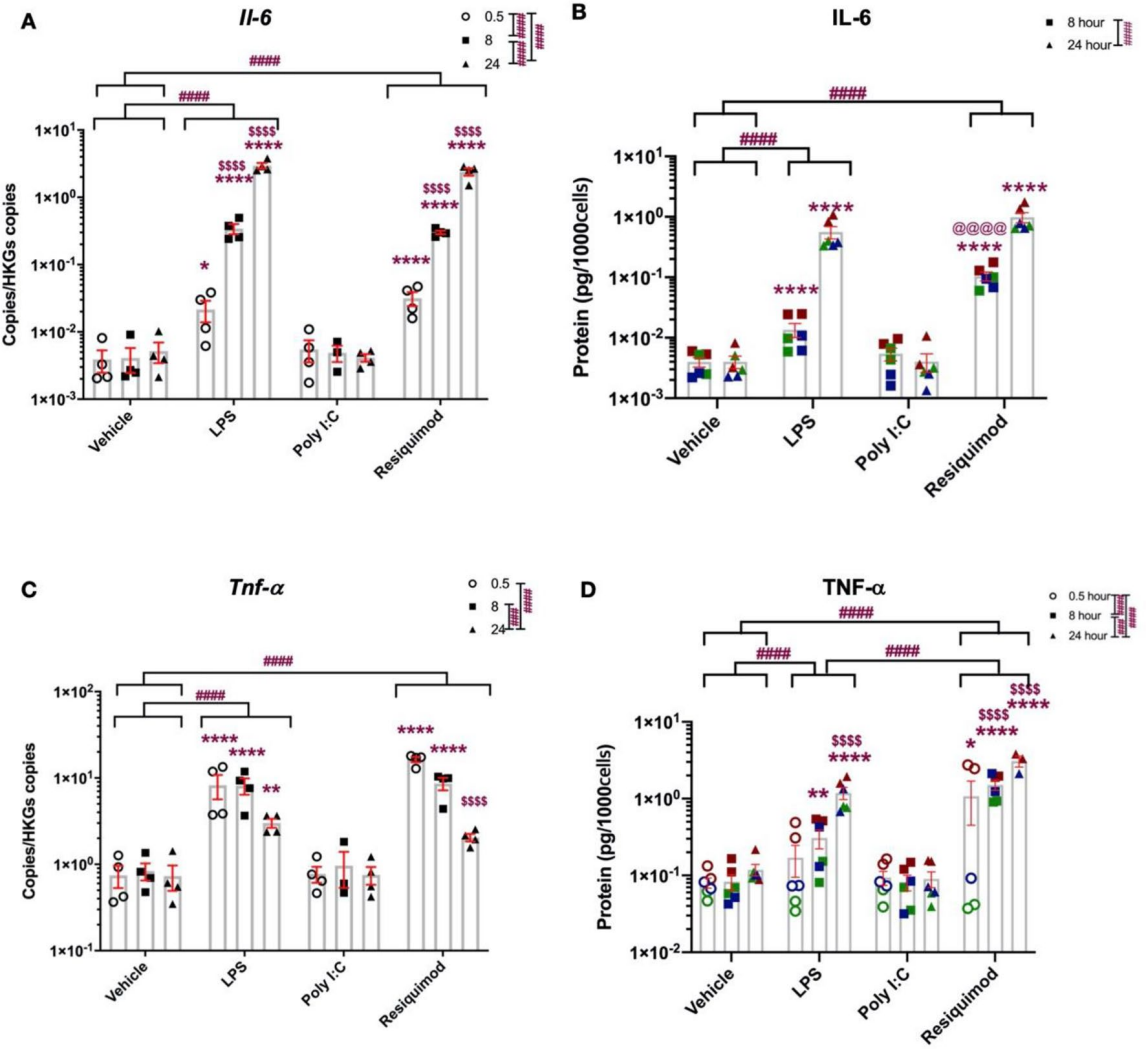
Distinct time courses of TNF and IL-6 induction after LPS and resiquimod. The microglial cells were cultured in serum-free medium overnight, stimulated by LPS (50 ng/ml), poly I:C (100 ng/ml), or resiquimod (3 μM), and RNA extracted and the culture media stored after the times indicated. **A** *Il-6* mRNA showed meaningful changes from 8 h stimulation with LPS and resiquimod but not with poly I:C. **B** IL-6 release increased statistically from 8 and 24 h stimulation with LPS and resiquimod. 0.5 h stimulation data were below quantification limit. **C** *Tnf* mRNA was maximally increased at 0.5 h after stimulation with LPS or resiquimod. **D** TNF release was significantly increased from 8 h and this level continued to increase. The individual data points are shown along with mean ± SEM. The colour represent passages of the cells. Data were analysed by two-way ANOVA (RT-qPCR); or three-way ANOVA (ELISA), Tukey post hoc test (*n* = 4–6 independent samples; *p ≤ 0.05, **p ≤ 0.005, ***p ≤ 0.001, ****p ≤ 0.0001 vs. vehicle control at same stimulation time; ^$^*p* ≤ 0.05, ^$$^*p* ≤ 0.005, ^$$$^*p* ≤ 0.001, ^$$$$^*p* ≤ 0.0001 vs. 0.5 h stimulation within the same treatment group; ^#^*p* ≤ 0.05, ^##^*p* ≤ 0.005, ^###^*p* ≤ 0.001, ^####^*p* ≤ 0.0001 overall effect Tukey comparisons; ^@^*p* ≤ 0.05, ^@@^*p* ≤ 0.005, ^@@@^*p* ≤ 0.001, ^@@@@^*p* ≤ 0.0001 LPS vs. resiquimod in a same stimulation time Tukey comparisons). (*n* = 4–6/group). Details of ANOVA *F* values and *p* values and of Fisher-Pitman permutation testing are provided in Supplementary Table 2

To explore other consequences of the immune challenge, the cells were stimulated for three different time points, 0.5, 8, 24 h in serum-free conditions, and cytokine and chemokine responses were measured, at both mRNA and protein level. *Il-6* mRNA significantly increased after LPS and resiquimod stimulation, however no changes were detected with poly I:C (Fig. 4A). *Il-6* mRNA levels continuously increased to 24 h. *Tnf* mRNA showed the peak stimulation at 0.5 h, after which its level gradually decreased (Fig. 4C). Again, poly I:C did not make any meaningful changes. Interestingly, IL-6 protein only showed detectable changes after 8 h; 0.5 h levels were below the quantification limit (data not shown), even though mRNA levels were raised from 0.5 h (Fig. 4B). TNF protein was significantly increased at 8 and 24 h (Fig. 4D). Thus whilst both *TNF* and *Il-6* genes are induced rapidly after TLR stimulation, *Il-6* mRNA levels continue to increase over 24 h, whereas *Tnf* mRNA levels start to decline. However, the levels of both proteins released from the cells rises over the 24 h period. At the concentrations used, resiquimod produced a greater release of TNF protein, compared to LPS (Fig. 4).

**Fig. 5:**
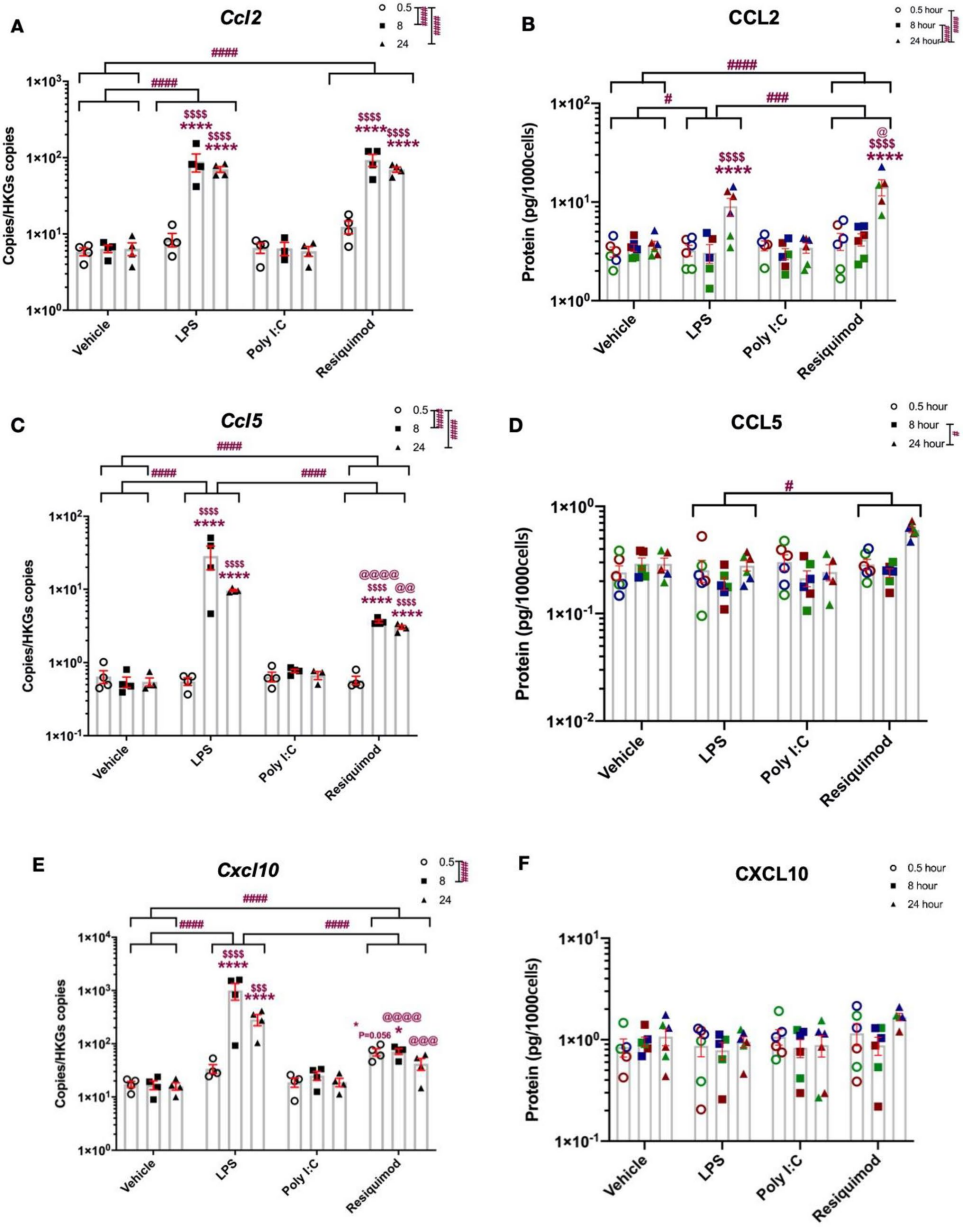
Distinct chemokine induction after LPS and resiquimod. The microglial cells were cultured in serum-free medium overnight, stimulated by LPS (50 ng/ml), poly I:C (100 ng/ml),or resiquimod (3 μM for the times indicated. **A** *Ccl2* mRNA levels were increased by LPS and resiquimod after 8 h stimulation but not by poly I:C. **B** CCL2 protein level showed meaningful changes with LPS and resiquimod stimulation at 24 h. **C** *Ccl5* mRNA showed considerable changes after 8 h resiquimod stimulation and its level remained increased till 24 h. LPS challenge induced an even greater increase in *Ccl5* mRNA levels from 8 and 24 h. **D** CCL5 protein levels did not change. **E** *Cxcl10* mRNA increased from 8 h after stimulation with LPS. Resiquimod showed a lesser induction from 0.5 h and its level was no different from control levels by 24 h. **F** CXCL10 protein levels did not change at any timepoints with any treatments. The individual data points are shown along with mean ± SEM. The colour represent passages of the cells. The data were analysed by two-way ANOVA (RT-qPCR); three-way ANOVA (ELISA), Tukey post hoc test (*n* = 4–6 independent samples; *p ≤ 0.05, **p ≤ 0.005, ***p ≤ 0.001, ****p ≤ 0.0001 vs. vehicle control at same stimulation time; ^$^*p* ≤ 0.05, ^$$^*p* ≤ 0.005, ^$$$^*p* ≤ 0.001, ^$$$$^*p* ≤ 0.0001 vs. 0.5 h stimulation within the same treatment group; ^#^*p* ≤ 0.05, ^##^*p* ≤ 0.005, ^###^*p* ≤ 0.001, ^####^*p* ≤ 0.0001 vs. overall effect Tukey comparisons; ^@^*p* ≤ 0.05, ^@@^*p* ≤ 0.005, ^@@@^*p* ≤ 0.001, ^@@@@^*p* ≤ 0.0001 LPS vs. resiquimod in a same stimulation time Tukey comparisons). (*n* = 4–6/group). Details of ANOVA *F* values and *p* values and of Fisher-Pitman permutation testing are provided in Supplementary Table 2

In contrast to pro-inflammatory cytokines, the time courses of changes in chemokine expression were almost consistent. *Ccl2, Ccl5* and *Cxcl10* mRNA levels robustly increased from 8 h and their levels remained at roughly the same level until 24 h (Fig. 5A,C,E). Thus the induction of these chemokine mRNAs showed a different temporal profile from both *TNF* and *Il-6*, with no further increase after 8 h. Responses to LPS and resiquimod were generally similar, but interestingly, *Cxcl10* mRNA showed measurable changes at 0.5 h with resiquimod but not LPS (Fig. 5E). Despite the LPS effect on *Cxcl10* mRNA starting more slowly, there was a much greater response to LPS by 24 h. The same greater response to LPS compared to resiquimod was seen with *Ccl5* mRNA (Fig. 5C). CCL2 protein release broadly reflected mRNA level changes even though it showed a later induction time point (Fig. 5B). CCL5 and CXCL10 protein levels, despite the mRNAs changing over time, did not show any increases (Fig. 5D,F). Our results suggest that bacterial infection will result in relatively greater chemokine (CCL5, CXCL10) release, and relatively less cytokine (IL-6, TNFα) release, from microglia, compared to single-stranded virus infection.These findings emphasise that protein level does not always reflect mRNA level, and other factors may influence the amount of protein that is detectable. Moreover, the nature of the immune response will clearly be different, depending on the pathogen.

### Effect of MAPK Inhibitors on Cytokine and Chemokine Induction

**Fig. 6:**
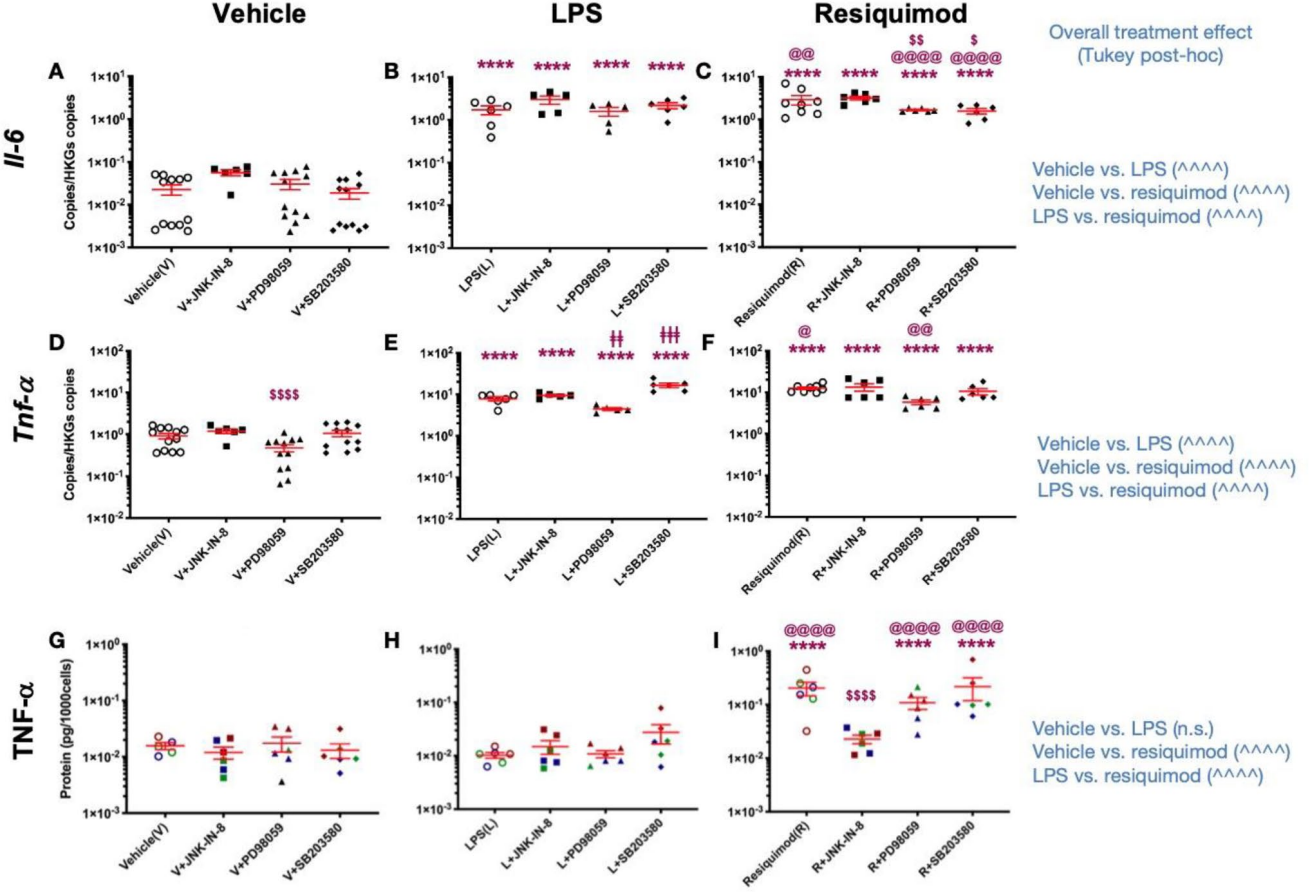
Pro-inflammatory cytokines are selectively regulated by MAPKs in microglia after LPS and resiquimod. The microglial cells were cultured in the serum-free medium and left overnight. The cells were stimulated by LPS (50 ng/ml) or resiquimod (3 μM) for 8 h and MAPK inhibitors, JNK (JNK-IN-8, JNK inhibitor, 1 μM), ERK (PD98059, ERK inhibitor, 40 μM), or p38 (SB203580, p38 inhibitor, 5 μM), or vehicle, were added before the mimetics. **A**–**C** *Il-6* mRNAs did not show any MAPKs’ inhibitor effects compare to vehicle or LPS only condition. ERK and p38 inhibition suppressed *Il-6* mRNA production when it combined with resiquimod. **D**–**F** *Tnf* mRNA did not any statistically significance of LPS or resiquimod with MAPKs’ inhibitors*.* **G**–**I** TNF-a proteins were only changed by JNK inhibitor with resiquimod. Absolute quantification was performed via RT-qPCR and the data were normalised to *Gapdh* and *Henmt1*. Total amount of protein (per 1000 cells) was presented on graphs and its amount was measured by ELISA. The individual data points are shown along with mean ± SEM. The colour represents passages of the cells. The data were analysed by three-way ANOVA, Tukey post hoc test (*n* = 5–12 independent samples; *p ≤ 0.05, **p ≤ 0.005, ***p ≤ 0.001, ****p ≤ 0.0001 vs. vehicle under the same inhibitor; ^$^*p* ≤ 0.05, ^$$^*p* ≤ 0.005, ^$$$^*p* ≤ 0.001, ^$$$$^*p* ≤ 0.0001 vs. vehicle alone within the same immune mimetic group; ^@^*p* ≤ 0.05, ^@@^*p* ≤ 0.005, ^@@@^p ≤ 0.001, ^@@@@^*p* ≤ 0.0001 vs. LPS+ a same inhibitor; ^^^*p* ≤ 0.05, ^^^^*p* ≤ 0.005, ^^^^^*p* ≤ 0.001, ^^^^^^*p* ≤ 0.0001 overall treatment effects; ^ǂ^*p* ≤ 0.05, ^ǂǂ^*p* ≤ 0.005, ^ǂǂǂ^*p* ≤ 0.001, ^ǂǂǂǂ^*p* ≤ 0.0001 LPS vs. LPS+PD98059 and LPS vs. SB203580 within LPS treatment group one-way ANOVA Bonferroni post hoc test). (*n* = 5–12/group). Details of ANOVA *F* values and *p* values are provided in Supplementary Information

To determine the effect of MAP kinase inhibition on cytokine and chemokine secretion, the cells were pre-treated with MAPK inhibitors, JNK-IN-8, PD98059, or SB203580, and then stimulated with LPS or resiquimod. After 8 h, LPS and resiquimod induced *Il-6* mRNA, and minor effects of MAPK inhibition were seen under resiquimod stimulation (Fig. 6A–C). IL-6 release with LPS or resiquimod was not detectable (data not shown). In contrast to *Il-6* mRNA, *Tnf* mRNA showed MAPK inhibition effects. The levels of *Tnf* mRNA were altered by ERK inhibition with vehicle and LPS (F(3,18) = 24.33, *p* < 0.0001; LPS vs. LPS+PD98059, *p* = 0.0079; LPS vs, LPS+SB203580, *p* = 0.0002, Bonferroni post hoc) but with resiquimod, however, JNK inhibition did not cause any alterations of *Tnf* mRNA (Fig. 6D–F). In spite of the alteration of *TNF* mRNA levels, TNF protein did not reflect these changes (Fig. 6G,H). In contrast to the lack of effect of JNK inhibition on *Tnf* mRNA induction by resiquimod, the increased TNF protein release was clearly inhibited (Fig. 6I). These results emphasise further the complex relationship between mRNA induction and protein release, and the distinct signalling pathways recruited in microglial cells by TLR4 and TLR7 stimulation (see Supplementary Figure 3).

**Fig. 7:**
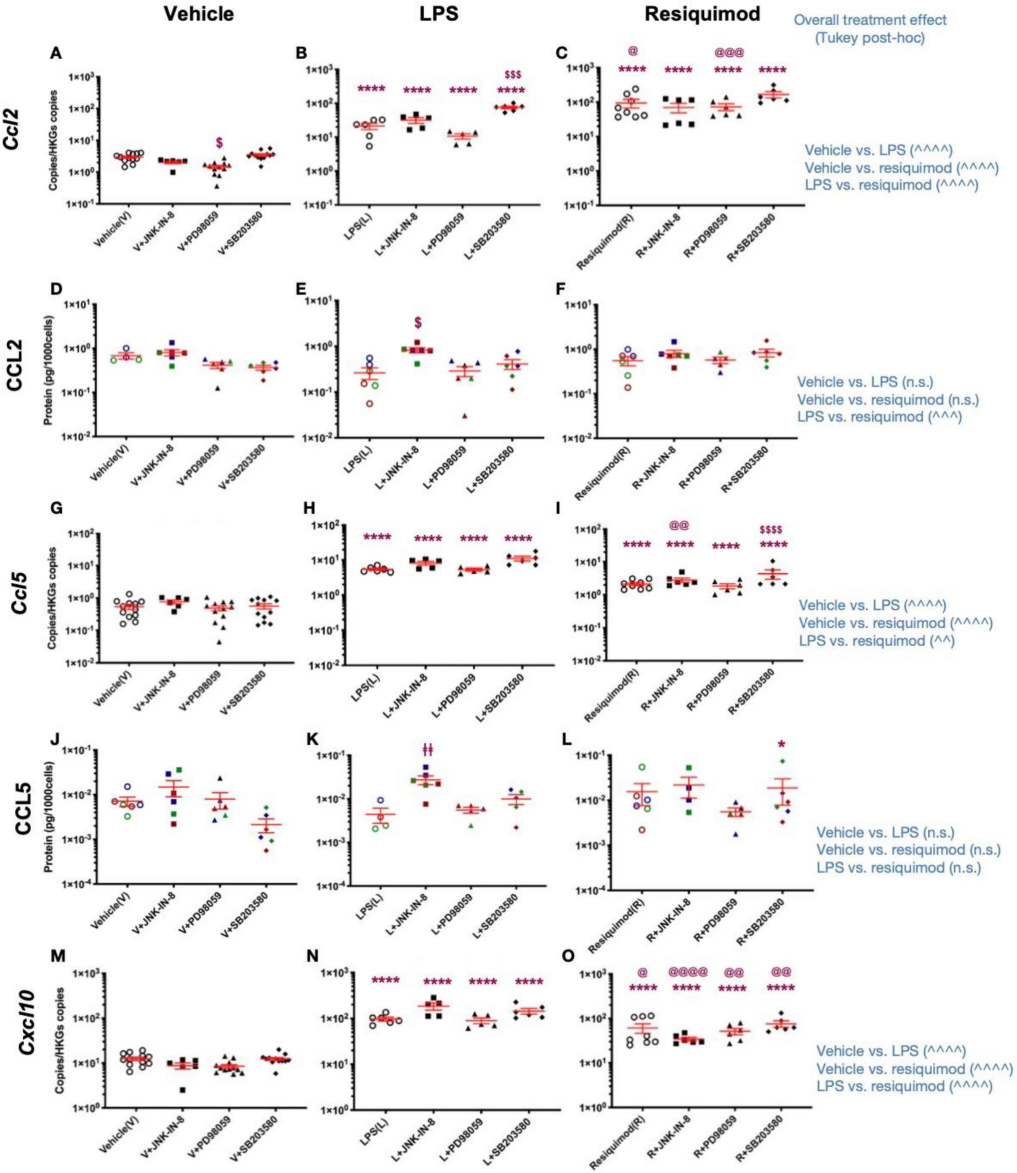
MAPKs selectively affect inflammatory chemokine in microglia after immune challenges. The cells were cultured in the serum-free medium and left overnight. The cells were stimulated by LPS (50 ng/ml) or resiquimod (3 μM) for 8 h and MAPK inhibitors, JNK (JNK-IN-8, JNK inhibitor, 1 μM), ERK (PD98059, ERK inhibitor, 40 μM), or p38 (SB203580, p38 inhibitor, 5 μM), were added before the mimetics. **A**–**C** *Ccl2* mRNA, **D**–**F** CCL2 protein, **G**–**I** *Ccl5* mRNA, **J**–**L** CCL5 protein, **M**–**O** *Cxcl10* mRNA. Absolute quantification was performed via RT-qPCR and the data were normalised to *Gapdh* and *Henmt1*. Total amount of protein (per 1000 cells) is shown. The individual data points are shown along with mean ± SEM. The colour represents passages of the cells. The data were analysed by three-way ANOVA, Tukey post hoc test (*n* = 5–12 independent samples; *p ≤ 0.05, **p ≤ 0.005, ***p ≤ 0.001, ****p ≤ 0.0001 vs. vehicle under a same inhibitor; ^$^*p* ≤ 0.05, ^$$^*p* ≤ 0.005, ^$$$^*p* ≤ 0.001, ^$$$$^*p* ≤ 0.0001 vs. vehicle alone within the same immune mimetic group; ^@^*p* ≤ 0.05, ^@@^*p* ≤ 0.005, ^@@@^*p* ≤ 0.001, ^@@@@^*p* ≤ 0.0001 vs. LPS + the same inhibitor; ^^^*p* ≤ 0.05, ^^^^*p* ≤ 0.005, ^^^^^*p* ≤ 0.001, ^^^^^*p* ≤ 0.0001 overall treatment effects; ^ǂ^*p* ≤ 0.05, ^ǂǂ^*p* ≤ 0.005, ^ǂǂǂ^*p* ≤ 0.001, ^ǂǂǂǂ^*p* ≤ 0.0001 LPS vs. LPS + JNK-IN-8 within LPS treatment group one-way ANOVA Bonferroni post hoc test). (*n* = 5–12/group). Details of ANOVA *F* values and *p* values are provided in Supplementary Information

Similarly complex regulation was observed with pro-inflammatory chemokines after MAPK inhibition. Under the vehicle condition, MAPK inhibition effects on *Ccl2* mRNA and CCL2 protein were not detected (Fig. 7A, D), but there was other evidence for JNK involvement in the regulation of CCL2 release, in that JNK inhibition increased the stimulatory effect of LPS, but not resiquimod (Fig. 7E, F). LPS stimulation increased *Ccl2* mRNA levels, which was further enhanced with p38 inhibition (Fig. 7B); however, this change was also not detectable in protein level (Fig. 7E). As observed previously, LPS produced a greater induction of *Ccl5* and *Cxcl10* mRNA than resiquimod. *Ccl5* mRNA was not affected by any MAPK inhibitor (Fig. 7G–I). CCL5 protein levels were not significantly changed (Fig. 7J–L). However, JNK inhibition with LPS induced CCL5 protein levels (F(3,16) = 7.917, *p* = 0.0018; LPS vs. LPS+JNK-IN-8, *p* = 0.0014; Bonferroni post hoc) and its secretion pattern is similar to CCL2 protein (Fig. 7E, K). Interestingly, p38 inhibition increased the *Ccl5* mRNA and CCL5 protein response to resiquimod (Fig. 7I, L). *Cxcl10* mRNA was not meaningfully changed by any MAPK inhibition (Fig. 7M–O). CXCL10 protein levels were below the threshold for detection (data not shown). These observations may suggest that MAPKs are not crucial, but selectively participate in chemokine induction during immune responses of microglia (see Supplementary Figure 3).

Many previous studies of the effects of JNK inhibition on the cytokine or chemokine responses in immune cells use the JNK inhibitor SP600125, which is fairly, but not completely selective for JNKs (Bain et al. 2007). When we compare the effects of SP600125 (at a widely used and putatively JNK-selective concentration) with the highly selective inhibitor JNK-IN-8 used here (Zhang et al. 2012) we observed some very clear differences between them (Supplementary Figure 3).

## Discussion

Through a direct comparison of the effects and mechanisms of different immune stimuli, we have found that the microglial cells studied here have very little response to TLR3 stimulation, distinct responses to TLR4 and TLR7 activation, and also a complex and distinct contribution of MAP kinase pathways to the regulation of the immune response.

### Lack of Response to polyIC

We detected clear MAP kinase/cytokine/chemokine responses to relatively low concentrations of LPS and resiquimod, with induction of all of the cytokines/chemokines measured. However, the general lack of responsiveness to polyI:C was surprising. The poly I:C employed clearly acts as a TLR3 stimulant, as we have previously observed substantial in vivo systemic immune responses to this batch of poly I:C in mice (Openshaw et al. 2019). The concentration of poly I:C used here in vitro—100 ng/ml—has previously been reported to activate p38 and JNK signalling in primary rat microglial cultures (de Oliveira et al. 2016), and to induce a large enhancement of cytokine release from microglia and other cells (Remels et al. 1990; Ribes et al. 2010; Peltier et al. 2010; Lehmann et al. 2012) via TLR3 stimulation. However, cytokine induction and JNK activation with a much higher (500x) poly I:C concentration are not mediated by TLR3 (Town et al. 2006).

Consistent with our data, many studies have reported a lack of response, or very low sensitivity, to poly I:C, in microglial cells (Olson and Miller 2004; Zuiderwijk‐Sick et al. 2007; Lobo‐Silva et al. 2017), and in peripheral blood mononuclear cells, where the cytokine and chemokine responses to poly I:C are 100–1000-fold smaller than the responses to TLR4 or TLR7/8 (2.5 μM resiquimod) stimulation (Ghosh et al. 2006; Monguió-Tortajada et al. 2018).

The expression of TLR3 in SIM-A9 cells was substantially lower than that of TLR4 and TLR7. Primary primate microglial cells also express very low levels of TLR3 (Zuiderwijk‐Sick et al. 2007). A possible explanation for the discrepant findings on poly I:C sensitivity may be that TLR3 expression is highly dynamic, with marked transcriptional induction depending on the degree of prior activation of the immune cells (Olson and Miller 2004; Town et al. 2006; Monguió-Tortajada et al. 2018). It seems likely that the SIM-A9 cells under our conditions are in a quasi-resting condition, with low basal *Tlr3* mRNA expression, possibly reflecting the lack of extensive and prolonged isolation procedures used for primary cultures, or viral imortalisation as in other microglial cell lines. Our data therefore support a growing awareness that sensitivity to TLR3 stimulation of microglia in their resting state may be rather limited.

### Relative Responses to TLR4 and TLR7 Stimulation—MAP Kinase Activation

Exposure of SIM-A9 microglial cells to LPS or resiquimod led to a rapid and pronounced phosphorylation of JNKs and p38s, with JNK activation being especially dramatic. Phosphorylation (activation) of ERKs 1 and 2 (p44 and p42 ERK) appeared to occur after a slight delay relative to the phosphorylation of JNKs and p38 MAP kinases. This is consistent with a report that primary microglial cells show ERK activation peaking at 20–30 min after LPS exposure (Bhat et al. 1998). However, the clear ERK response to LPS in SIM-A9 cells as well as primary microglia is interesting, since the BV-2 mouse microglial cell line reportedly shows very little ERK activation at any time after LPS exposure (Watters et al. 2002). This probably reflects high basal levels of ERK activity, as these cells are immortalised by activation of this pathway (Blasi et al. 1990).

### Relative Responses to TLR4 and TLR7 Stimulation—Cytokines

Agonists at TLR7/8 are relatively poorly studied in microglial cells, in comparison to TLR3 or TLR4 agonists. We found that resiquimod was a powerful stimulant of cytokine and chemokine release, consistent with its effects on MAP kinase phosphorylation. Expression of TLR8, was relatively low, suggesting that the effects of resiquimod are mediated via TLR7 rather than TLR8. The strong responses that we observe following TLR7 activation, as compared to activation of TLR3, or even TLR4, are consistent with evidence that stimulation of cytokine (TNF) release from primary primate microglial cells is also especially sensitive to TLR7/8 agonists (Zuiderwijk‐Sick et al. 2007). There is controversy with primary mouse microglia, which have been reported to be relatively unresponsive to TLR7 stimulation, compared to TLR4 stimulation, (Lee et al. 2016), or especially sensitive to the TLR7 stimulation (Butchi et al. 2008). These discrepancies again may relate to the degree of prior sensitisation of the cells. Our data are also consistent with data from mouse macrophages (Pauls et al. 2013) where 3 μM resiquimod produces much greater increases in *Tnf-a* mRNA compared to 100 ng/ml LPS.

It is interesting to note that *Il6* mRNA and IL-6 protein induction develops relatively slowly, over 24 h, in contrast *Tnf-a* mRNA and TNF protein induction, which is detected in 0.5 h, even though they are in the same functional group (pro-inflammatory cytokines). This has been noted in other systems (Eskay et al. 1990) and so is not unique to microglia.

### Relative Responses to TLR4 and TLR7 Stimulation—Chemokine Induction

Cxcl10 mRNA induction after resiquimod is remarkably fast (mRNA is elevated after 0.5 h), but then declines, in contrast to the slower but more sustained time course after LPS, and indeed the slower but more sustained time course after resiquimod for Ccl2 and Ccl5 mRNAs. This is similar to the rapid induction of *TNF* mRNA, and, in terms of mechanism, at least partially reflects fast pre-mRNA splicing for these two genes (Hao and Baltimore 2013). Similar effects have been noted in vivo, where induction of Cxcl10 in vivo by *Toxoplasma Gondii* infection is faster than induction of Ccl2/Ccl5 (Khan et al. 2000).

For most of the cytokine/chemokines monitored, the degree of induction by LPS and resiquimod was roughly equivalent, at the concentrations used. However, it was noticeable that the profile of the response differed, with LPS producing a greater induction of *Ccl5* and *Cxcl10* mRNAs, whilst resiquimod produced a greater induction of *Tnf*, *Il6* and *Ccl2* mRNAs (Figs. 4, 5, 6, 7), indicative of distinct signalling pathways downstream of TLR4 and TLR7.

### Effects of p38 Kinase Inhibition

Inhibition of p38 tended to facilitate nitrite production after TLR4 stimulation. In previous reports, SB203580 is often observed to diminish iNOS induction, or nitrite production in microglial cells (Pyo et al. 1998; Bhat et al. 1998; Watters et al. 2002; Han et al. 2002; Wen et al. 2011). However, in macrophage cells, selective p38 inhibition *increases* nitrite release following LPS exposure (Lahti et al. 2002, 2006), due to enhanced JNK signalling, normally dampened by a p38-mediated destabilisation of JNK. Since higher concentrations of SB203580 (30 μM) inhibit nitrite production by a mechanism unrelated to p38 inhibition (Lahti et al. 2002), this may provide an explanation for some of the discrepant reports.

As predicted from other studies (Tessaro et al. 2017; Rutault et al. 2001), p38 inhibition reduced TNF release. However, we found no reduction in *TNF* mRNA with p38 inhibition. In fact, mRNA levels were increased with p38 inhibition after LPS, but not resiquimod, treatment. This resembles the recently reported increased *TNF* mRNA levels after p38 inhibition in epithelial cells, reflecting loss of a p38-mediated transcriptional induction of the *TNF* mRNA destabiliser tristetraprolin (Shah et al. 2016). Our data suggest the operation of a similar mechanism in microglia. These actions of p38 are likely to be very time-dependent, as p38 also rapidly suppresses tristetraprolin-mediated destabilisation of its target mRNAs via phosphorylation events (Ronkina et al. 2019; Prabhala et al. 2015). The overt post-transcriptional effects of p38 inhibition on TNF release probably reflect actions on TRIF (Gais et al. 2010), or MAPKAP-K2 (Kotlyarov et al. 1999; Tiedje et al. 2014).

It is interesting to note that the enhancing effect of p38 inhibition on *TNF* mRNA is also seen with *Ccl2* and *Ccl5* mRNA, as *Ccl2* and *Ccl5* mRNAs are also known to be tristetraprolin targets (Tu et al. 2019; Schichl et al. 2009; Ishmael et al. 2008). Moreover, this p38 action is clearly both time- and pathogen-specific, and yet implies some commonality in the regulation of this group of genes.

### Effect of JNK and ERK Inhibition—Cytokine and Nitric Oxide Production

Inhibition of JNK or ERK suppressed immune mimetic-stimulated nitric oxide and TNF release. The effect on nitric oxide was more pronounced with LPS, whilst the effect on TNF was more pronounced with resiquimod. ERK inhibition, but not JNK inhibition, also decreased *TNF* mRNA induction by stimulation of both TLR4 and TLR7. Induction of IL-6 was less dramatic than TNF, but this may reflect the time point chosen, and the slower induction of *Il-6* mRNA compared to *TNF* mRNA (Fig. 3).

The suppression of microglial nitric oxide release, following exposure to LPS or resiquimod, by inhibition of ERK or JNK pathways, has been shown in a number of previous studies, both in microglia, and in other immune response cells (Bhat et al. 1998; Pyo et al. 1998; Lahti et al. 2003; Wang et al. 2004; Yu et al. 2017). ERK and JNK are also generally reported to mediate increased TNF release in response to LPS (Swantek et al. 1997; Scherle et al. 1998; Zhu et al. 2000; Wang et al. 2004, 2010; Sánchez-Tilló et al. 2007). The lack of suppression of TNF release by inhibition of JNK pathways reported here, following exposure to LPS, was not predicted; the more so considering that inhibiting JNK does suppress the response to resiquimod (summarised in Supplementary Figure 3). To understand the role of JNK following TLR7, but not TLR4, stimulation, it is worth noting that TRAF6 may contribute to signalling by the former but not the latter (Loniewski et al. 2007), and that TRAF6 mediates JNK activation in various cell types (Kobayashi et al. 2001; Wan et al. 2004).

### Effect of JNK and ERK Kinase Inhibition—Chemokine Production

Selective inhibition of JNK enhanced the release of CCL2 by LPS, but not by resiquimod. Again this illustrates the selectivity with which distinct TLRs recruit distinct signalling pathways, and reveals that JNK can suppress CCL2 release.

We noted above that the enhancing effect of p38 inhibition on *Tnf*, *Ccl2* and *Ccl5* mRNAs implied some commonality in the regulation of this group of genes. However, whilst *Ccl5* showed a larger induction by TLR4 compared to TLR7 stimulation, this was also true for *Cxcl10*, but not for *Ccl2* (Figs. 5, 7). JNK inhibition further enhances *Cxcl10* responses to LPS but not resiquimod (as indeed it enhances TNF response to resiquimod but not LPS). This effect of JNK is despite the larger LPS responses noted above, suggesting additional (JNK-mediated) mechanisms may be recruited to dampen the powerful *Cxcl10* response to TLR4 stimulation.

There are a number of contradictory findings concerning the extent to which ERK, p38 and JNK mediate induction of CCL2, CCL5 and CXCL10 release from microglia and other immune cells (Aversa et al. 2004; Nakamichi et al. 2005; Bandow et al. 2012). Some of these discrepancies may reflect differential control of mRNA versus protein, as noted above.

Much of the confusion in the literature regarding the effects of JNK inhibition on the immune response no doubt reflects the use of SP600125, which is fairly, but not completely selective for JNKs. Indeed, when we compare the effects of SP600125 (at a putatively JNK-selective concentration) with the highly selective inhibitor JNK-IN-8 (Zhang et al. 2012) we observed some fairly dramatic differences (Supplementary Figure 3). SP6001265, at a concentration sufficient to inhibit JNKs, will also inhibit various kinases including SGK1 and PKD1 (Bain et al. 2007), which likely contribute to the variable results obtained in other studies.

It is notable from our data that p38 regulates Ccl2 and Ccl5 at the mRNA level whilst JNK acts at the post-transcriptional level. Similar effects are seen with TNF (Supplementary Figures 3). The dramatically differing effects on mRNA vs protein of JNK and p38, whilst supported by prior reports, are not easily understood in terms of the functional significance. It may be that the post-transcriptional control allows a “fine tuning” of the courser control provided by transcriptional regulation (rheostat vs switch), and it has been proposed that a wider dynamic range is generally observed for protein as compared to mRNA (Vogel and Marcotte 2012). Our results therefore implicate JNK in this fine tuning of microglial responses, and p38 rather in broad control. Indeed, where the magnitude of the response to TLR4 and TLR7 stimulation differs, this seems to be due to the selective activation of a p38-mediated suppression of mRNA levels in the lower of the responses (Supplementary Figure 3).

## Conclusions

The data show that TLR3 stimulation has little effect on MAP kinase activation or cytokine/chemokine responses in resting microglial cells. TLR4 and TLR7 stimulation produces clear effects, but the profile of cytokine/chemokine induction differs between them, as do the signalling pathways involved. With the exception of TNF release, inhibition of ERK, JNK or p38 had surprisingly little effect to suppress cytokine or chemokine induction, and in some specific instances actually enhanced the effects of LPS or resiquimod.

The question remains, if MAP kinase pathways are exerting subtle modulatory effects on the microglial inflammatory response, then what signalling pathway is responsible for the main gene induction events downstream of TLR stimulation ? The most likely candidate is the NF-kappaB pathway, which a wide range of evidence implicates in microglial activation alongside MAPK pathways (Kaminska et al. 2016). Indeed, there is evidence that LPS is much more effective than poly I:C at activating NF-kappaB in human macrophages (Reimer et al. 2008)**.** It is also worth considering the possibility that simultaneous combined inhibition of all three major MAP kinase pathways (ERK, JNK and p38), might produce a qualitatively greater suppression of inflammatory mediator induction than individual inhibition of each component. However, current evidence suggests that interactions between the different kinase cascades tend to be negative feedback cross-regulation rather than synergistic downstream actions (Junttila et al. 2008; Boutros et al. 2008), and where inhibitors of different MAPK pathways have been applied in combination, synergistic inhibitory effects have not been observed (Dolivo et al. 2019).

The effect of p38 inhibition to enhance rather than suppress mRNA induction of some inflammatory mediators may be highly relevant for the current development of p38 inhibitors as therapeutic agents for neurodegenerative and psychiatric disease (Scheltens et al. 2018; Wittenberg et al. 2019). Furthermore, our results suggest that infection with single-stranded viruses will produce much greater microglial TNF release than bacterial infection. In any situation where JNK activity is elevated (as for example in genetic predisposition to schizophrenia (Winchester 2012), this induction of TNFα will be even further increased. This may be highly relevant for understanding the basis of the increased risk of schizophrenia caused by antenatal viral infections.

## Supplementary Information

Below is the link to the electronic supplementary material.

Supplementary file1 (PDF 953 kb)

## Data Availability

The datasets generated during and/or analysed during the current study are available from the corresponding author on reasonable request.

## References

[CR1] Autret A, Martin-Latil S, Brisac C, Mousson L, Colbère-Garapin F, Blondel B (2008) Early phosphatidylinositol 3-kinase/Akt pathway activation limits poliovirus-induced JNK-mediated cell death. J Virol 82(7):3796–3802. 10.1128/JVI.02020-07 18216097 10.1128/JVI.02020-07 PMC2268503

[CR2] Aversa TGD, Yu KOA, Berman JW (2004) Expression of chemokines by human fetal microglia after treatment with the human immunodeficiency virus type 1 protein Tat. J Neurovirol 10(2):86–97. 10.1080/13550280490279807 15204927 10.1080/13550280490279807

[CR3] Bain J, Plater L, Elliott M, Shpiro N, Hastie CJ, Mclauchlan H, Klevernic I, Arthur JSC, Alessi DR, Cohen P (2007) The selectivity of protein kinase inhibitors: a further update. Biochem J 408(3):297–315. 10.1042/BJ20070797 17850214 10.1042/BJ20070797 PMC2267365

[CR4] Bandow K, Kusuyama J, Shamoto M, Kakimoto K, Ohnishi T, Matsuguchi T (2012) LPS-induced chemokine expression in both MyD88-dependent and-independent manners is regulated by Cot/Tpl2-ERK axis in macrophages. FEBS Lett 586(10):1540–1546. 10.1016/j.febslet.2012.04.018 22673523 10.1016/j.febslet.2012.04.018

[CR5] Baufeld C, O’Loughlin E, Calcagno N, Madore C, Butovsky O (2018) Differential contribution of microglia and monocytes in neurodegenerative diseases. J Neural Transm 125(5):809–826. 10.1007/s00702-017-1795-7 29063348 10.1007/s00702-017-1795-7 PMC7255107

[CR6] Beckham JD, Goody RJ, Clarke P, Bonny C, Tyler KL (2007) Novel strategy for treatment of viral central nervous system infection by using a cell-permeating inhibitor of c-Jun N-terminal kinase. J Virol 81(13):6984–6992. 10.1128/JVI.00467-07 17475657 10.1128/JVI.00467-07 PMC1933289

[CR7] Bhat NR, Zhang P, Lee JC, Hogan EL (1998) Extracellular signal-regulated kinase and p38 subgroups of mitogen-activated protein kinases regulate inducible nitric oxide synthase and tumor necrosis factor-α gene expression in endotoxin-stimulated primary glial cultures. J Neurosci 18(5):1633–1641. 10.1523/JNEUROSCI.18-05-01633.1998 9464988 10.1523/JNEUROSCI.18-05-01633.1998 PMC6792636

[CR8] Blasi E, Barluzzi R, Bocchini V, Mazzolla R, Bistoni F (1990) Immortalization of murine microglial cells by a v-raf/v-myc carrying retrovirus. J Neuroimmunol 27(2–3):229–237. 10.1016/0165-5728(90)90073-V 2110186 10.1016/0165-5728(90)90073-v

[CR9] Boutros T, Chevet E, Metrakos P (2008) Mitogen-activated protein (MAP) kinase/MAP KINASE phosphatase regulation: roles in cell growth, death, and cancer. Pharmacol Rev 60(3):261–310. 10.1124/pr.107.00106 18922965 10.1124/pr.107.00106

[CR10] Bsibsi M, Ravid R, Gveric D, van Noort JM (2002) Broad expression of toll-like receptors in the human central nervous system. J Neuropathol Exp Neurol 61(11):1013–1021. 10.1093/jnen/61.11.1013 12430718 10.1093/jnen/61.11.1013

[CR11] Butchi NB, Pourciau S, Du M, Morgan TW, Peterson KE (2008) Analysis of the neuroinflammatory response to TLR7 stimulation in the brain: comparison of multiple TLR7 and/or TLR8 agonists. J Immunol 180(11):7604–7612. 10.4049/jimmunol.180.11.7604 18490763 10.4049/jimmunol.180.11.7604

[CR12] Butovsky O, Jedrychowski MP, Moore CS, Cialic R, Lanser AJ, Gabriely G, Koeglsperger T, Dake B, Wu PM, Doykan CE, Fanek Z, Liu L, Chen Z, Rothstein JD, Ransohoffl RM, Gygi SP, Antel JP, Weiner HL (2014) Identification of a unique TGF-beta dependent molecular and functional signature in microglia. Nat Neurosci 17(1):131–143. 10.1038/nn.3599 24316888 10.1038/nn.3599 PMC4066672

[CR13] Cliffe Anna R, Arbuckle Jesse H, Vogel Jodi L, Geden Matthew J, Rothbart Scott B, Cusack Corey L, Strahl Brian D, Kristie Thomas M, Deshmukh M (2015) Neuronal stress pathway mediating a histone methyl/phospho switch is required for herpes simplex virus reactivation. Cell Host Microbe 18(6):649–658. 10.1016/j.chom.2015.11.007 26651941 10.1016/j.chom.2015.11.007 PMC4681005

[CR14] Coffey ET (2014) Nuclear and cytosolic JNK signalling in neurons. Nat Rev Neurosci 15(5):285–299. 10.1038/nrn3729 24739785 10.1038/nrn3729

[CR15] Crews L, Patrick C, Achim C, Everall I, Masliah E (2009) Molecular pathology of neuro-AIDS (CNS-HIV). Int J Mol Sci 10(3):1045–1063. 10.3390/ijms10031045 19399237 10.3390/ijms10031045 PMC2672018

[CR16] Das A, Kim SH, Arifuzzaman S, Yoon T, Chai JC, Lee YS, Park KS, Jung KH, Chai YG (2016) Transcriptome sequencing reveals that LPS-triggered transcriptional responses in established microglia BV2 cell lines are poorly representative of primary microglia. J Neuroinflamm 13(1):182. 10.1186/s12974-016-0644-1 10.1186/s12974-016-0644-1 PMC4940985 27400875

[CR17] Davis RJ (2000) Signal transduction by the JNK group of MAP kinases. Cell 103(2):239–252. 10.1016/s0092-8674(00)00116-1 11057897 10.1016/s0092-8674(00)00116-1

[CR18] de Oliveira ACP, Yousif NM, Bhatia HS, Hermanek J, Huell M, Fiebich BL (2016) Poly (I: C) increases the expression of mPGES-1 and COX-2 in rat primary microglia. J Neuroinflamm 13(1):11. 10.1186/s12974-015-0473-7 10.1186/s12974-015-0473-7 PMC4717620 26780827

[CR19] Del Villar K, Miller CA (2004) Down-regulation of DENN/MADD, a TNF receptor binding protein, correlates with neuronal cell death in Alzheimer’s disease brain and hippocampal neurons. Proc Natl Acad Sci USA 101(12):4210–4215. 10.1073/pnas.0307349101 15007167 10.1073/pnas.0307349101 PMC384720

[CR20] Dolivo DM, Larson SA, Dominko T (2019) Crosstalk between mitogen-activated protein kinase inhibitors and transforming growth factor-β signaling results in variable activation of human dermal fibroblasts. Int J Mol Med 43(1):325–335. 10.3892/ijmm.2018.3949 30365043 10.3892/ijmm.2018.3949 PMC6257852

[CR21] Dong C, Davis RJ, Flavell RA (2002) MAP kinases in the immune response. Annu Rev Immunol 20(1):55–72. 10.1146/annurev.immunol.20.091301.131133 11861597 10.1146/annurev.immunol.20.091301.131133

[CR22] Efthymiou AG, Goate AM (2017) Late onset Alzheimer’s disease genetics implicates microglial pathways in disease risk. Mol Neurodegener 12(1):43. 10.1186/s13024-017-0184-x 28549481 10.1186/s13024-017-0184-x PMC5446752

[CR23] Eskay RL, Grino M, Chen HT (1990) Interleukins, signal transduction, and the immune system-mediated stress response. Circulating regulatory factors and neuroendocrine function. Springer, Boston, pp 331–343. 10.1007/978-1-4684-5799-5_21 10.1007/978-1-4684-5799-5_21 2239431

[CR24] Fillman SG, Cloonan N, Catts VS, Miller LC, Wong J, McCrossin T, Cairns M, Weickert CS (2013) Increased inflammatory markers identified in the dorsolateral prefrontal cortex of individuals with schizophrenia. Mol Psychiatry 18(2):206–214. 10.1038/mp.2012.110 22869038 10.1038/mp.2012.110

[CR25] Gais P, Tiedje C, Altmayr F, Gaestel M, Weighardt H, Holzmann B (2010) TRIF signaling stimulates translation of TNF-α mRNA via prolonged activation of MK2. J Immunol 184(10):5842–5848. 10.4049/jimmunol.0902456 20375303 10.4049/jimmunol.0902456

[CR26] Ghosh TK, Mickelson DJ, Fink J, Solberg JC, Inglefield JR, Hook D, Gupta SK, Gibson S, Alkan SS (2006) Toll-like receptor (TLR) 2–9 agonists-induced cytokines and chemokines: I. Comparison with T cell receptor-induced responses. Cell Immunol 243(1):48–57. 10.1016/j.cellimm.2006.12.002 17250816 10.1016/j.cellimm.2006.12.002

[CR27] Guilding C, McNair K, Stone TW, Morris BJ (2007) Restored plasticity in a mouse model of neurofibromatosis type 1 via inhibition of hyperactive ERK and CREB. Eur J Neurosci 25(1):99–105. 10.1111/j.1460-9568.2006.05238.x 17241271 10.1111/j.1460-9568.2006.05238.x

[CR28] Han I-O, Kim K-W, Ryu JH, Kim W-K (2002) p38 mitogen-activated protein kinase mediates lipopolysaccharide, not interferon-γ,-induced inducible nitric oxide synthase expression in mouse BV2 microglial cells. Neurosci Lett 325(1):9–12. 10.1016/S0304-3940(02)00218-5 12023055 10.1016/s0304-3940(02)00218-5

[CR29] Hao S, Baltimore D (2013) RNA splicing regulates the temporal order of TNF-induced gene expression. Proc Natl Acad Sci USA 110(29):11934–11939. 10.1073/pnas.1309990110 23812748 10.1073/pnas.1309990110 PMC3718113

[CR30] Hemmi H, Kaisho T, Takeuchi O, Sato S, Sanjo H, Hoshino K, Horiuchi T, Tomizawa H, Takeda K, Akira S (2002) Small anti-viral compounds activate immune cells via the TLR7 MyD88-dependent signaling pathway. Nat Immunol 3(2):196–200 11812998 10.1038/ni758

[CR31] Henn A, Lund S, Hedtjärn M, Schrattenholz A, Pörzgen P, Leist M (2009) The suitability of BV2 cells as alternative model system for primary microglia cultures or for animal experiments examining brain inflammation. Altex 26(2):83–94. 10.14573/altex.2009.2.83 19565166 10.14573/altex.2009.2.83

[CR32] Herron JW, Nerurkar L, Cavanagh J (2018) Neuroimmune biomarkers in mental illness. Biomarkers in psychiatry. Springer, Cham, pp 45–78. 10.1007/7854_2018_45 10.1007/7854_2018_45 29626340

[CR33] Hong S, Lee EE, Martin AS, Soontornniyomkij B, Soontornniyomkij V, Achim CL, Reuter C, Irwin MR, Eyler LT, Jeste DV (2017) Abnormalities in chemokine levels in schizophrenia and their clinical correlates. Schizophr Res 181:63–69. 10.1016/j.schres.2016.09.019 27650194 10.1016/j.schres.2016.09.019 PMC5357211

[CR34] Ishmael FT, Fang X, Galdiero MR, Atasoy U, Rigby WFC, Gorospe M, Cheadle C, Stellato C (2008) Role of the RNA-binding protein tristetraprolin in glucocorticoid-mediated gene regulation. J Immunol 180(12):8342–8353. 10.4049/jimmunol.180.12.8342 18523301 10.4049/jimmunol.180.12.8342 PMC2505276

[CR35] Junttila MR, Li S-P, Westermarck J (2008) Phosphatase-mediated crosstalk between MAPK signaling pathways in the regulation of cell survival. FASEB J 22(4):954–965. 10.1096/fj.06-7859rev 18039929 10.1096/fj.06-7859rev

[CR36] Kaminska B, Mota M, Pizzi M (2016) Signal transduction and epigenetic mechanisms in the control of microglia activation during neuroinflammation. Biochim Biophys Acta 1862(3):339–351. 10.1016/j.bbadis.2015.10.026 26524636 10.1016/j.bbadis.2015.10.026

[CR37] Khan IA, MacLean JA, Lee FS, Casciotti L, DeHaan E, Schwartzman JD, Luster AD (2000) IP-10 is critical for effector T cell trafficking and host survival in Toxoplasma gondii infection. Immunity 12(5):483–494. 10.1016/S1074-7613(00)80200-9 10843381 10.1016/s1074-7613(00)80200-9

[CR38] Kobayashi N, Kadono Y, Naito A, Matsumoto K, Yamamoto T, Tanaka S, Ji I (2001) Segregation of TRAF6-mediated signaling pathways clarifies its role in osteoclastogenesis. EMBO J 20(6):1271–1280. 10.1093/emboj/20.6.1271 11250893 10.1093/emboj/20.6.1271 PMC145527

[CR39] Kotlyarov A, Neininger A, Schubert C, Eckert R, Birchmeier C, Volk H-D, Gaestel M (1999) MAPKAP kinase 2 is essential for LPS-induced TNF-α biosynthesis. Nat Cell Biol 1(2):94. 10.1038/10061 10559880 10.1038/10061

[CR40] Kuan CY, Yang DD, Roy DRS, Davis RJ, Rakic P, Flavell RA (1999) The Jnk1 and Jnk2 protein kinases are required for regional specific apoptosis during early brain development. Neuron 22(4):667–676. 10.1016/s0896-6273(00)80727-8 10230788 10.1016/s0896-6273(00)80727-8

[CR41] Lahti A, Kankaanranta H, Moilanen E (2002) P38 mitogen-activated protein kinase inhibitor SB203580 has a bi-directional effect on iNOS expression and NO production. Eur J Pharmacol 454(2–3):115–123. 10.1016/S0014-2999(02)02490-1 12421638 10.1016/s0014-2999(02)02490-1

[CR42] Lahti A, Jalonen U, Kankaanranta H, Moilanen E (2003) c-Jun NH2-terminal kinase inhibitor anthra (1, 9-cd) pyrazol-6 (2H)-one reduces inducible nitric-oxide synthase expression by destabilizing mRNA in activated macrophages. Mol Pharmacol 64(2):308–315. 10.1124/mol.64.2.308 12869635 10.1124/mol.64.2.308

[CR43] Lahti A, Sareila O, Kankaanranta H, Moilanen E (2006) Inhibition of p38 mitogen-activated protein kinase enhances c-Jun N-terminal kinase activity: implication in inducible nitric oxide synthase expression. BMC Pharmacol 6(1):5. 10.1186/1471-2210-6-5 16504051 10.1186/1471-2210-6-5 PMC1402273

[CR44] Lee J-W, Nam H, Yu S-W (2016) Systematic analysis of translocator protein 18 kDa (TSPO) ligands on toll-like receptors-mediated pro-inflammatory responses in microglia and astrocytes. Exp Neurobiol 25(5):262–268. 10.5607/en.2016.25.5.262 27790060 10.5607/en.2016.25.5.262 PMC5081472

[CR45] Lehmann SM, Krüger C, Park B, Derkow K, Rosenberger K, Baumgart J, Trimbuch T, Eom G, Hinz M, Kaul D (2012) An unconventional role for miRNA: let-7 activates toll-like receptor 7 and causes neurodegeneration. Nat Neurosci 15(6):827. 10.1038/nn.3113 22610069 10.1038/nn.3113

[CR46] Lobo-Silva D, Carriche GM, Castro AG, Roque S, Saraiva M (2017) Interferon-β regulates the production of IL-10 by toll-like receptor-activated microglia. Glia 65(9):1439–1451. 10.1002/glia.23172 28617991 10.1002/glia.23172 PMC7165667

[CR47] Loniewski KJ, Patial S, Parameswaran N (2007) Sensitivity of TLR4-and-7-induced NFκB1 p105-TPL2-ERK pathway to TNF-receptor-associated-factor-6 revealed by RNAi in mouse macrophages. Mol Immunol 44(15):3715–3723. 10.1016/j.molimm.2007.04.002 17507094 10.1016/j.molimm.2007.04.002

[CR48] Melief J, Sneeboer MAM, Litjens M, Ormel PR, Palmen S, Huitinga I, Kahn RS, Hol EM, de Witte LD (2016) Characterizing primary human microglia: a comparative study with myeloid subsets and culture models. Glia 64(11):1857–1868. 10.1002/glia.23023 27442614 10.1002/glia.23023

[CR49] Michaelis M, Kleinschmidt MC, Doerr HW, Cinatl J Jr (2007) Minocycline inhibits West Nile virus replication and apoptosis in human neuronal cells. J Antimicrob Chemother 60(5):981–986. 10.1093/jac/dkm307 17872917 10.1093/jac/dkm307

[CR50] Miller BJ, Buckley P, Seabolt W, Mellor A, Kirkpatrick B (2011) Meta-analysis of cytokine alterations in schizophrenia: clinical status and antipsychotic effects. Biol Psychiatry 70(7):663–671. 10.1016/j.biopsych.2011.04.013 21641581 10.1016/j.biopsych.2011.04.013 PMC4071300

[CR51] Monguió-Tortajada M, Franquesa M, Sarrias M-R, Borràs FE (2018) Low doses of LPS exacerbate the inflammatory response and trigger death on TLR3-primed human monocytes. Cell Death Dis 9(5):499. 10.1038/s41419-018-0520-2 29717111 10.1038/s41419-018-0520-2 PMC5931601

[CR52] Morris BJ, Pratt JA (2014) Novel treatment strategies for schizophrenia from improved understanding of genetic risk. Clin Genet 86(5):401–411. 10.1111/cge.12485 25142969 10.1111/cge.12485

[CR53] Mufson EJ, He B, Nadeem M, Perez SE, Counts SE, Leurgans S, Fritz J, Lah J, Ginsberg SD, Wuu J (2012) Hippocampal proNGF signaling pathways and β-amyloid levels in mild cognitive impairment and Alzheimer disease. J Neuropathol Exp Neurol 71(11):1018–1029. 10.1097/NEN.0b013e318272caab 23095849 10.1097/NEN.0b013e318272caab PMC3481187

[CR54] Nagamoto-Combs K, Kulas J, Combs CK (2014) A novel cell line from spontaneously immortalized murine microglia. J Neurosci Methods 233:187–198. 10.1016/j.jneumeth.2014.05.021 24975292 10.1016/j.jneumeth.2014.05.021 PMC4140094

[CR55] Nakamichi K, Saiki M, Sawada M, Takayama-Ito M, Yamamuro Y, Morimoto K, Kurane I (2005) Rabies virus-induced activation of mitogen-activated protein kinase and NF-κB signaling pathways regulates expression of CXC and CC chemokine ligands in microglia. J Virol 79(18):11801–11812. 10.1128/JVI.79.18.11801-11812.2005 16140757 10.1128/JVI.79.18.11801-11812.2005 PMC1212600

[CR56] Olson JK, Miller SD (2004) Microglia initiate central nervous system innate and adaptive immune responses through multiple TLRs. J Immunol 173(6):3916–3924. 10.4049/jimmunol.173.6.3916 15356140 10.4049/jimmunol.173.6.3916

[CR57] Openshaw RL, Kwon J, McColl A, Penninger JM, Cavanagh J, Pratt JA, Morris BJ (2019) JNK signalling mediates aspects of maternal immune activation: importance of maternal genotype in relation to schizophrenia risk. J Neuroinflamm 16(1):18. 10.1186/s12974-019-1408-5 10.1186/s12974-019-1408-5 PMC6350402 30691477

[CR58] Pagès G, Guérin S, Grall D, Bonino F, Smith A, Anjuere F, Auberger P, Pouysségur J (1999) Defective thymocyte maturation in p44 MAP kinase (Erk 1) knockout mice. Science 286(5443):1374. 10.1126/science.286.5443.1374 10558995 10.1126/science.286.5443.1374

[CR59] Pauls E, Nanda SK, Smith H, Toth R, Arthur JSC, Cohen P (2013) Two phases of inflammatory mediator production defined by the study of IRAK2 and IRAK1 knock-in mice. J Immunol 191(5):2717–2730. 10.4049/jimmunol.1203268 23918981 10.4049/jimmunol.1203268 PMC3849919

[CR60] Peltier DC, Simms A, Farmer JR, Miller DJ (2010) Human neuronal cells possess functional cytoplasmic and TLR-mediated innate immune pathways influenced by phosphatidylinositol-3 kinase signaling. J Immunol 184(12):7010–7021. 10.4049/jimmunol.0904133 20483728 10.4049/jimmunol.0904133 PMC2887731

[CR61] Prabhala P, Bunge K, Rahman MM, Ge Q, Clark AR, Ammit AJ (2015) Temporal regulation of cytokine mRNA expression by tristetraprolin: dynamic control by p38 MAPK and MKP-1. Am J Physiol Lung Cell Mol Physiol 308(9):L973–L980. 10.1152/ajplung.00219.2014 25724669 10.1152/ajplung.00219.2014

[CR62] Pyo H, Jou I, Jung S, Hong S, Joe E-h (1998) Mitogen-activated protein kinases activated by lipopolysaccharide and β-amyloid in cultured rat microglia. NeuroReport 9(5):871–874 9579682 10.1097/00001756-199803300-00020

[CR63] Reimer T, Brcic M, Schweizer M, Jungi TW (2008) poly(I:C) and LPS induce distinct IRF3 and NF-κB signaling during type-I IFN and TNF responses in human macrophages. J Leukoc Biol 83(5):1249–1257. 10.1189/jlb.0607412 18252870 10.1189/jlb.0607412

[CR64] Remels L, Fransen L, Huygen K, De Baetselier P (1990) Poly I: C activated macrophages are tumoricidal for TNF-alpha-resistant 3LL tumor cells. J Immunol 144(11):4477–4486 2111350

[CR65] Ribes S, Adam N, Ebert S, Regen T, Bunkowski S, Hanisch U-K, Nau R (2010) The viral TLR3 agonist poly (I: C) stimulates phagocytosis and intracellular killing of Escherichia coli by microglial cells. Neurosci Lett 482(1):17–20. 10.1016/j.neulet.2010.06.078 20599470 10.1016/j.neulet.2010.06.078

[CR66] Righi M, Mori L, Libero GD, Sironi M, Biondi A, Mantovani A, Donini SD, Ricciardi-Castagnoli P (1989) Monokine production by microglial cell clones. Eur J Immunol 19(8):1443–1448. 10.1002/eji.1830190815 2789141 10.1002/eji.1830190815

[CR67] Ronkina N, Shushakova N, Tiedje C, Yakovleva T, Tollenaere MAX, Scott A, Batth TS, Olsen JV, Helmke A, Bekker-Jensen SH, Clark AR, Kotlyarov A, Gaestel M (2019) The role of TTP phosphorylation in the regulation of inflammatory cytokine production by MK2/3. J Immunol 203(8):2291–2300. 10.4049/jimmunol.1801221 31527197 10.4049/jimmunol.1801221

[CR68] Rutault K, Hazzalin CA, Mahadevan LC (2001) Combinations of ERK and p38 MAPK inhibitors ablate tumor necrosis factor-α (TNF-α) mRNA induction evidence for selective destabilization of TNF-α transcripts. J Biol Chem 276(9):6666–6674. 10.1074/jbc.M005486200 11076936 10.1074/jbc.M005486200

[CR69] Sánchez-Tilló E, Comalada M, Xaus J, Farrera C, Valledor AF, Caelles C, Lloberas J, Celada A (2007) JNK1 Is required for the induction of Mkp1 expression in macrophages during proliferation and lipopolysaccharide-dependent activation. J Biol Chem 282(17):12566–12573. 10.1074/jbc.M609662200 17337450 10.1074/jbc.M609662200

[CR70] Scheltens P, Prins N, Lammertsma A, Yaqub M, Gouw A, Wink AM, Chu HM, van Berckel BNM, Alam J (2018) An exploratory clinical study of p38α kinase inhibition in Alzheimer’s disease. Ann Clin Transl Neurol 5(4):464–473. 10.1002/acn3.549 29687023 10.1002/acn3.549 PMC5899915

[CR71] Scherle PA, Jones EA, Favata MF, Daulerio AJ, Covington MB, Nurnberg SA, Magolda RL, Trzaskos JM (1998) Inhibition of MAP kinase kinase prevents cytokine and prostaglandin E2 production in lipopolysaccharide-stimulated monocytes. J Immunol 161(10):5681–5686 9820549

[CR72] Schichl YM, Resch U, Hofer-Warbinek R, de Martin R (2009) Tristetraprolin impairs NF-κB/p65 nuclear translocation. J Biol Chem 284(43):29571–29581. 10.1074/jbc.M109.031237 19654331 10.1074/jbc.M109.031237 PMC2785590

[CR73] Shah S, Mostafa MM, McWhae A, Traves SL, Newton R (2016) Negative feed-forward control of tumor necrosis factor (TNF) by tristetraprolin (ZFP36) is limited by the mitogen-activated protein kinase phosphatase, dual-specificity phosphatase 1 (DUSP1) implications for regulation by glucocorticoids. J Biol Chem 291(1):110–125. 10.1074/jbc.M115.697599 26546680 10.1074/jbc.M115.697599 PMC4697149

[CR74] Stansley B, Post J, Hensley K (2012) A comparative review of cell culture systems for the study of microglial biology in Alzheimer’s disease. J Neuroinflamm 9(1):115. 10.1186/1742-2094-9-115 10.1186/1742-2094-9-115 PMC3407712 22651808

[CR75] Swantek JL, Cobb MH, Geppert TD (1997) Jun N-terminal kinase/stress-activated protein kinase (JNK/SAPK) is required for lipopolysaccharide stimulation of tumor necrosis factor alpha (TNF-alpha) translation: glucocorticoids inhibit TNF-alpha translation by blocking JNK/SAPK. Mol Cell Biol 17(11):6274–6282. 10.1128/MCB.17.11.6274 9343388 10.1128/mcb.17.11.6274 PMC232478

[CR76] Swardfager W, Lanctôt K, Rothenburg L, Wong A, Cappell J, Herrmann N (2010) A meta-analysis of cytokines in Alzheimer’s disease. Biol Psychiat 68(10):930–941. 10.1016/j.biopsych.2010.06.012 20692646 10.1016/j.biopsych.2010.06.012

[CR77] Swatton JE, Sellers LA, Faull RLM, Holland A, Iritani S, Bahn S (2004) Increased MAP kinase activity in Alzheimer’s and down syndrome but not in schizophrenia human brain. Eur J Neurosci 19(10):2711–2719. 10.1111/j.1460-9568.2004.03365.x 15147305 10.1111/j.0953-816X.2004.03365.x

[CR78] Takahashi Y, Yu Z, Sakai M, Tomita H (2016) Linking activation of microglia and peripheral monocytic cells to the pathophysiology of psychiatric disorders. Front Cell Neurosci 10:144. 10.3389/fncel.2016.00144 27375431 10.3389/fncel.2016.00144 PMC4891983

[CR79] Tessaro FHG, Ayala TS, Nolasco EL, Bella LM, Martins JO (2017) Insulin influences LPS-Induced TNF-α and IL-6 release through distinct pathways in mouse macrophages from different compartments. Cell Physiol Biochem 42(5):2093–2104. 10.1159/000479904 28810254 10.1159/000479904

[CR80] Tiedje C, Holtmann H, Gaestel M (2014) The role of mammalian MAPK signaling in regulation of cytokine mRNA stability and translation. J Interferon Cytokine Res 34(4):220–232. 10.1089/jir.2013.0146 24697200 10.1089/jir.2013.0146

[CR81] Town T, Jeng D, Alexopoulou L, Tan J, Flavell RA (2006) Microglia recognize double-stranded RNA via TLR3. J Immunol 176(6):3804–3812. 10.4049/jimmunol.176.6.3804 16517751 10.4049/jimmunol.176.6.3804

[CR82] Tran EH, Azuma Y-T, Chen M, Weston C, Davis RJ, Flavell RA (2006) Inactivation of JNK1 enhances innate IL-10 production and dampens autoimmune inflammation in the brain. Proc Natl Acad Sci USA 103(36):13451–13456. 10.1073/pnas.0601155103 16938889 10.1073/pnas.0601155103 PMC1569184

[CR83] Trudler D, Farfara D, Frenkel D (2010) Toll-like receptors expression and signaling in glia cells in neuro-amyloidogenic diseases: towards future therapeutic application. Mediat Inflamm. 10.1155/2010/497987 10.1155/2010/497987 PMC2913815 20706642

[CR84] Tu Y, Wu X, Yu F, Dang J, Wang J, Wei Y, Cai Z, Zhou Z, Liao W, Li L, Zhang Y (2019) Tristetraprolin specifically regulates the expression and alternative splicing of immune response genes in HeLa cells. BMC Immunol 20(1):13. 10.1186/s12865-019-0292-1 31046669 10.1186/s12865-019-0292-1 PMC6498542

[CR85] Vogel C, Marcotte EM (2012) Insights into the regulation of protein abundance from proteomic and transcriptomic analyses. Nat Rev Genet 13(4):227. 10.1038/nrg3185 22411467 10.1038/nrg3185 PMC3654667

[CR86] Wan J, Sun L, Mendoza JW, Chui YL, Huang DP, Chen ZJ, Suzuki N, Suzuki S, Yeh W-C, Akira S (2004) Elucidation of the c-Jun N-terminal kinase pathway mediated by Epstein-Barr virus-encoded latent membrane protein 1. Mol Cell Biol 24(1):192–199. 10.1128/MCB.24.1.192-199.2004 14673155 10.1128/MCB.24.1.192-199.2004 PMC303354

[CR87] Wang M-J, Jeng K-CG, Kuo J-S, Chen H-L, Huang H-Y, Chen W-F, Lin S-Z (2004) c-Jun N-terminal kinase and to a lesser extent, p38 mitogen-activated protein kinase regulate inducible nitric oxide synthase expression in hyaluronan fragments-stimulated BV-2 microglia. J Neuroimmunol 146(1–2):50–62. 10.1016/j.jneuroim.2003.10.034 14698847 10.1016/j.jneuroim.2003.10.034

[CR88] Wang M-J, Huang H-Y, Chen W-F, Chang H-F, Kuo J-S (2010) Glycogen synthase kinase-3β inactivation inhibits tumor necrosis factor-α production in microglia by modulating nuclear factor κB and MLK3/JNK signaling cascades. J Neuroinflamm 7(1):99. 10.1186/1742-2094-7-99 10.1186/1742-2094-7-99 PMC3022821 21194439

[CR89] Watters JJ, Sommer JA, Pfeiffer ZA, Prabhu U, Guerra AN, Bertics PJ (2002) A differential role for the mitogen-activated protein kinases in lipopolysaccharide signaling the MEK/ERK pathway is not essential for nitric oxide and interleukin 1β production. J Biol Chem 277(11):9077–9087. 10.1074/jbc.M104385200 11786532 10.1074/jbc.M104385200

[CR90] Wen J, Ribeiro R, Zhang Y (2011) Specific PKC isoforms regulate LPS-stimulated iNOS induction in murine microglial cells. J Neuroinflamm 8(1):38. 10.1186/1742-2094-8-38 10.1186/1742-2094-8-38 PMC3110130 21510893

[CR91] Winchester CL, Ohzeki H, Vouyiouklis DA, Thompson R, Penninger JM, Yamagami K, Norrie JD, Hunter R, Pratt JA, Morris BJ (2012) Converging evidence that sequence variations in the novel candidate gene MAP2K7 (MKK7) are functionally associated with schizophrenia. Hum Mol Genet 21(22):4910–4921. 10.1093/hmg/dds331 22899651 10.1093/hmg/dds331

[CR92] Wittenberg GM, Stylianou A, Zhang Y, Sun Y, Gupta A, Jagannatha PS, Wang D, Hsu B, Curran ME, Khan S (2019) Effects of immunomodulatory drugs on depressive symptoms: A mega-analysis of randomized, placebo-controlled clinical trials in inflammatory disorders. Mol Psychiatry. 10.1038/s41380-019-0471-8 31427751 10.1038/s41380-019-0471-8 PMC7244402

[CR93] Yarza R, Vela S, Solas M, Ramirez MJ (2016) c-Jun N-terminal kinase (JNK) signaling as a therapeutic target for Alzheimer’s disease. Front Pharmacol 6:321. 10.3389/fphar.2015.00321 26793112 10.3389/fphar.2015.00321 PMC4709475

[CR94] Yeh FL, Hansen DV, Sheng M (2017) TREM2, microglia, and neurodegenerative diseases. Trends Mol Med 23(6):512–533. 10.1016/j.molmed.2017.03.008 28442216 10.1016/j.molmed.2017.03.008

[CR95] Yu LE, Lai CL, Lee CT, Wang JY (2017) Highly electronegative low-density lipoprotein L5 evokes microglial activation and creates a neuroinflammatory stress via toll-like receptor 4 signaling. J Neurochem 142(2):231–245. 10.1111/jnc.14053 28444734 10.1111/jnc.14053

[CR96] Zhang XY, Zhou DF, Zhang PY, Wu GY, Cao LY, Shen YC (2002) Elevated interleukin-2, interleukin-6 and interleukin-8 serum levels in neuroleptic-free schizophrenia: association with psychopathology. Schizophr Res 57(2):247–258. 10.1016/S0920-9964(01)00296-1 12223256 10.1016/s0920-9964(01)00296-1

[CR97] Zhang T, Inesta-Vaquera F, Niepel M, Zhang J, Ficarro SB, Machleidt T, Xie T, Marto JA, Kim N, Sim T (2012) Discovery of potent and selective covalent inhibitors of JNK. Chem Biol 19(1):140–154. 10.1016/j.chembiol.2011.11.010 22284361 10.1016/j.chembiol.2011.11.010 PMC3270411

[CR98] Zhao X, Guo Y, Jiang C, Chang Q, Zhang S, Luo T, Zhang B, Jia X, Hung M-C, Dong C, Lin X (2017) JNK1 negatively controls antifungal innate immunity by suppressing CD23 expression. Nat Med 23(3):337–346. 10.1038/nm.4260 28112734 10.1038/nm.4260 PMC5592785

[CR99] Zhu W, Downey JS, Gu J, Di Padova F, Gram H, Han J (2000) Regulation of TNF expression by multiple mitogen-activated protein kinase pathways. J Immunol 164(12):6349–6358. 10.4049/jimmunol.164.12.6349 10843689 10.4049/jimmunol.164.12.6349

[CR100] Zuiderwijk-Sick EA, Van der Putten C, Bsibsi M, Deuzing IP, De Boer W, Persoon-Deen C, Kondova I, Boven LA, Van Noort JM, t Hart BA (2007) Differentiation of primary adult microglia alters their response to TLR8-mediated activation but not their capacity as APC. Glia 55(15):1589–1600. 10.1002/glia.20572 17823968 10.1002/glia.20572

