# Supplementary material for: Differential Effects of Toll-Like Receptor Activation and Differential Mediation by MAP Kinases of Immune Responses in Microglial Cells
Source: Cell Mol Neurobiol. 2021 Jul 23;42(8):2655–71. doi: 10.1007/s10571-021-01127-x (PMC9560989; doi:10.1007/s10571-021-01127-x)
Supplement: Supplementary file 1 — Supplementary file1 (PDF 953 kb) [file 10571_2021_1127_MOESM1_ESM.pdf]

Supplementary Information:

Contents:

Supplementary Figure 1 - LPS time-course experiment

Supplementary Figure 2 – Poly I:C time-course experiment

Supplementary Figure 3 - Schematic interaction between MAPKs and immune molecules in microglia

Supplementary Figure 4 – SP600125 vs JNK-IN-8

Supplementary Figure 5 – Example full-length scans of western blots

Supplementary Table 1 – RT-qPCR primer sequences and amplicon sizes

Supplementary Table 2 – Details of statistical analysis output

Supplementary Figure 1

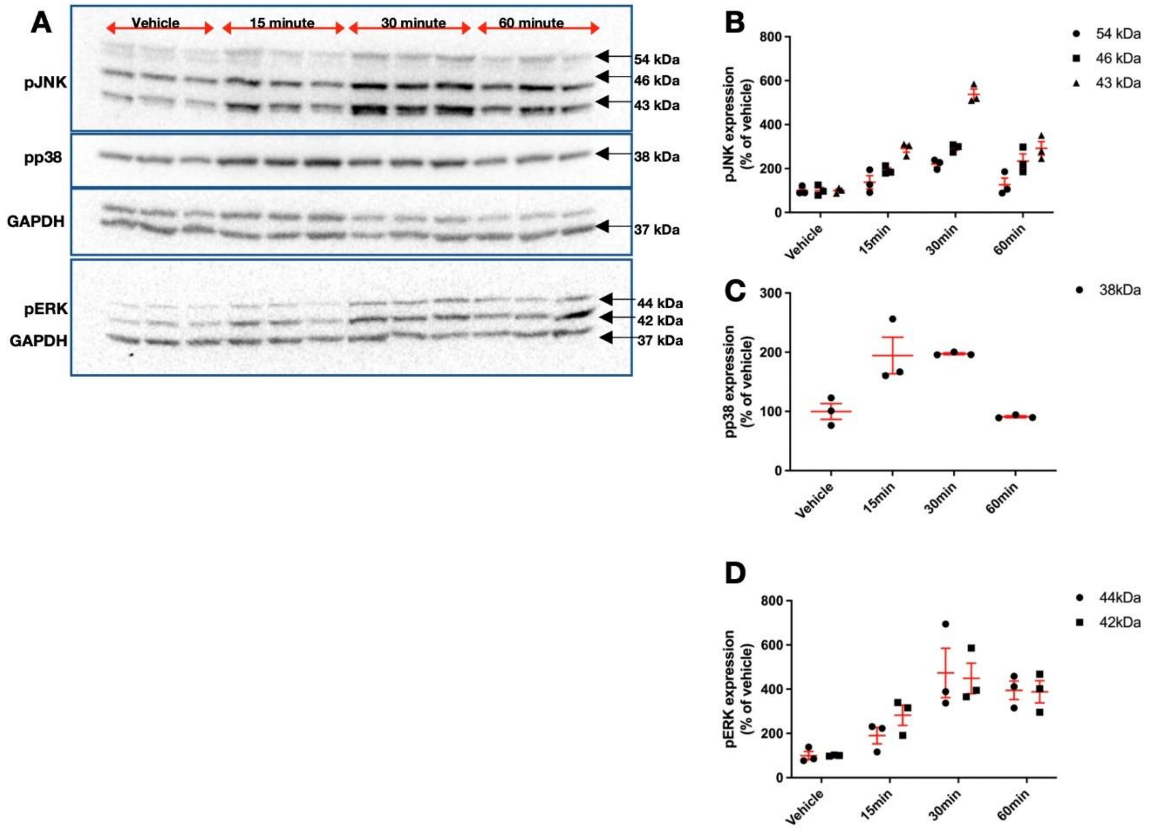

LPS time-course experiment

Figure 1. LPS requires longer exposure time to induce pMAPKs. The SIM-A9 cells were cultured in serum-free condition and left overnight before treatment. The cells were culture with LPS (50ng/ml) for 15 minutes, 30 minutes and 60 minutes. Because of the size of proteins, pMAPK was measured in two separated membranes, pJNKs and pp38 were in a same membrane, pERKs were observed in the other membrane. (A) After 30 minutes stimulation, all MAPKs, JNK, ERK, and p38 were phosphorylated more than vehicle. The upper bands in the GAPDH image were pp38 bands. (B,C) The increased level of pMAPK in 30 minutes was clear, although 15 min stimulation might change significantly depending on isoforms e.g. 46 kDa and 43 kDa pJNKs. Individual dots expression relative vehicle ± SEM for three independent experiments. The data were log transformed and analysed by two way ANOVA, Tukey comparison (pJNK, pERK) and by one-way ANOVA, Bonferroni comparison (pp38).

Supplementary Figure 2

Poly I:C timecourse

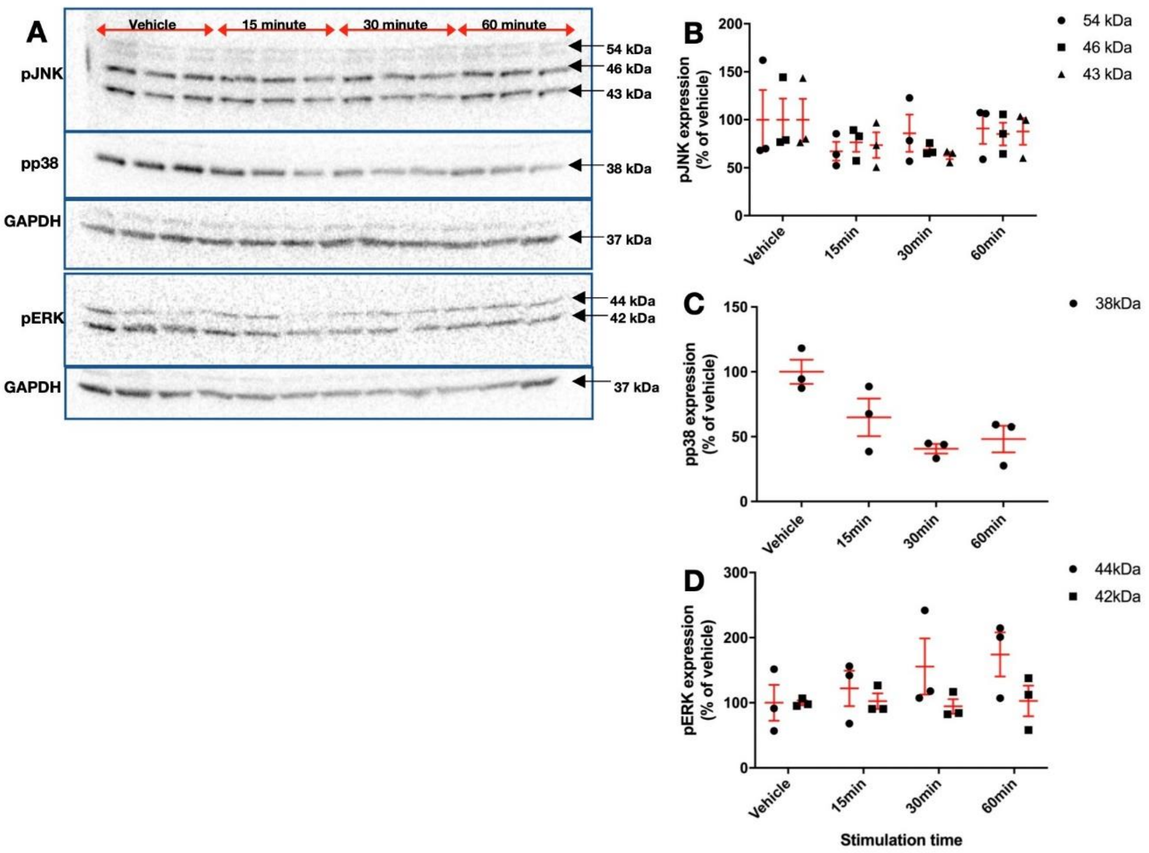

Figure 2. Poly I:C does not induce pMAPKs. The microglia were passaged with serum-free medium and left overnight before use. The cells were treated by Poly I:C (100 ng/ml) for 15 minutes, 30 minutes and 60 minutes. Depends on the size of proteins, pMAPKs were measured in two separated membranes, pJNK and pp38 were in a same membrane, pERK was observed in the other membrane. (A) Poly I:C did not change the levels of pJNK and pERK, but the phosphorylation of p38 was suppressed by the treatment. (B-D) The level of pp38 was considerably reduced at 30 minutes exposure, while pJNK and pERK were not significantly different from the vehicle condition over all exposure time. Individual dots expression relative vehicle ± SEM. N=3/group. The data were log transformed and analysed by two way ANOVA, two-way ANOVA, Tukey comparison (pJNK, pERK) and by one-way ANOVA, Bonferroni comparison (pp38).

Supplementary Figure 3

Schematic interaction between MAPKs and immune molecules in microglia

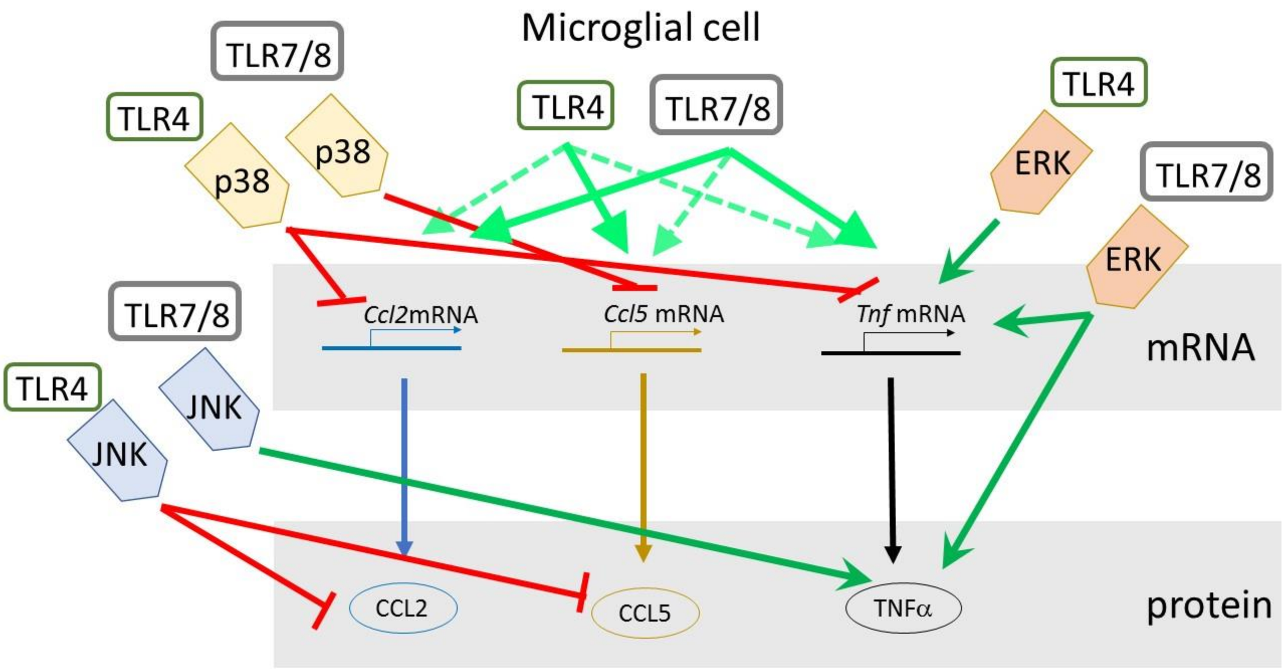

Figure 3. Complex connection between transcription and translation with MAPK signalling pathway. When the microglia are stimulated by TLR4 and TLR7, MAPKs are activated to respond to environmental challenges and will result in releasing cytokines and chemokines. However, MAPK signalling pathways do not always work as positive modulators. For example, p38 dominantly acts as a suppressor, but ERKs work as an inducer. Our results suggest that MAPKs signalling pathways participate in immune reaction in the microglia, although in a complex and stimulus-specific manner.

Supplementary Figure 4

SP600125 vs. JNK-IN-8

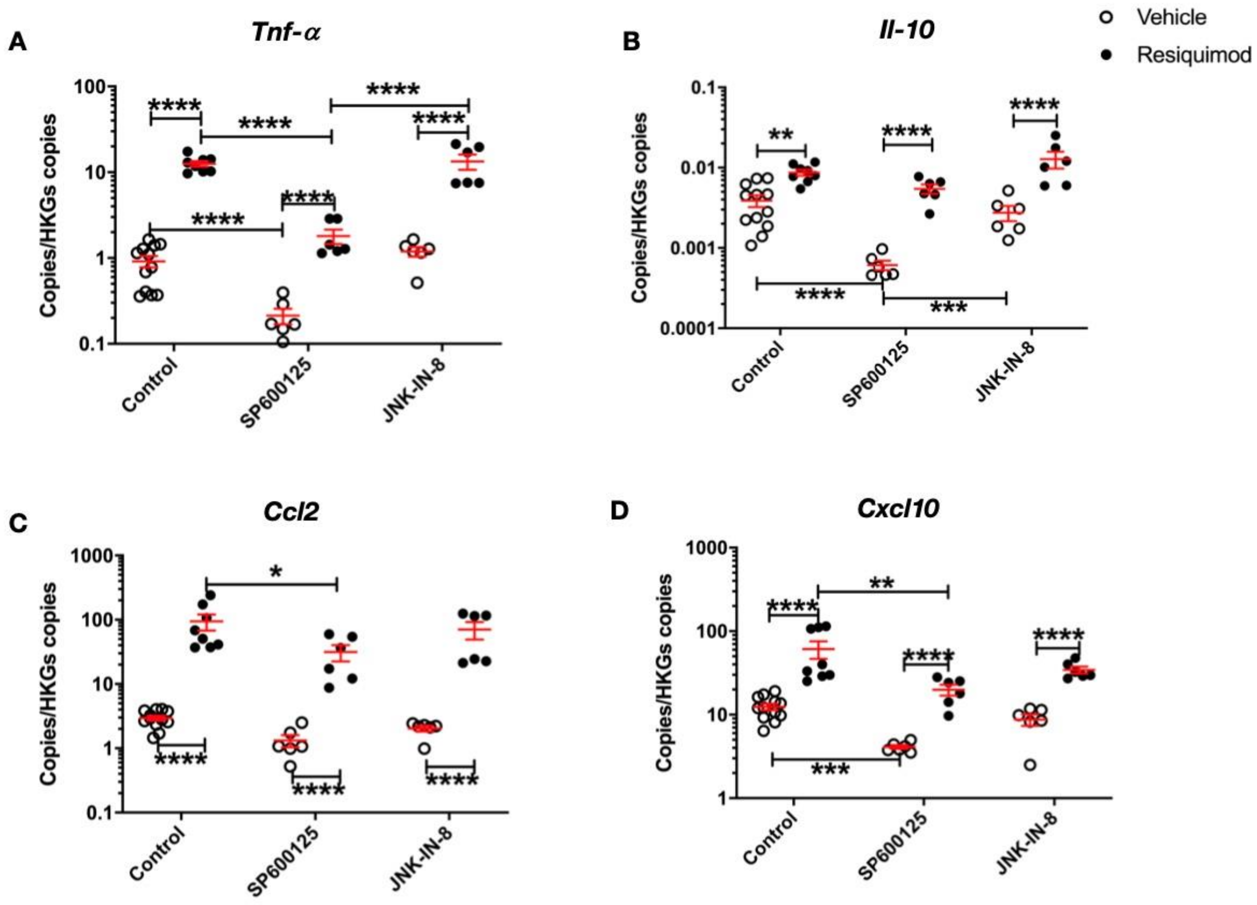

Figure 4. SP600125 inhibits not only JNKs but also other signalling kinases. The microglial cells were cultured in the serum-free medium and left overnight. The cells were stimulated by resiquimod (3μM) for 8 hour and two types of JNK inhibitors, JNK-IN-8 (Sigma, 1μM, 3hours ahead), or SP600125 (Enzo, 5μM, 0.5hours ahead) were added before resiquimod. (A) Tnf-α mRNA was not affected by JNK-IN-8, but SP600125 decreasing mRNA levels in both, vehicle and resiquimod conditions. (B) SP600125 down-regulated Il-10 mRNA under vehicle although SP600125 did not affect *Il-10* mRNA level under resiquimod condition. (C) SP600125 affected *Ccl2* mRNA in only resiquimod condition (D) *Cxcl10* mRNA was not affected by JNK-IN-8, but SP600125 suppressed *Cxcl10* mRNA in both conditions compared to control. The individual data points are shown along with mean ± SEM. The data were log transformed and analysed by two-way ANOVA, tukey post-hoc test (n=5-6 independent samples; *p≤0.05, **p≤0.005, ***p≤0.001, ****p≤0.0001 Tukey comparison)

Supplementary Figure 5 – Example full-length scans of western blots, with examples showing parallel electrophoresis of size markers.

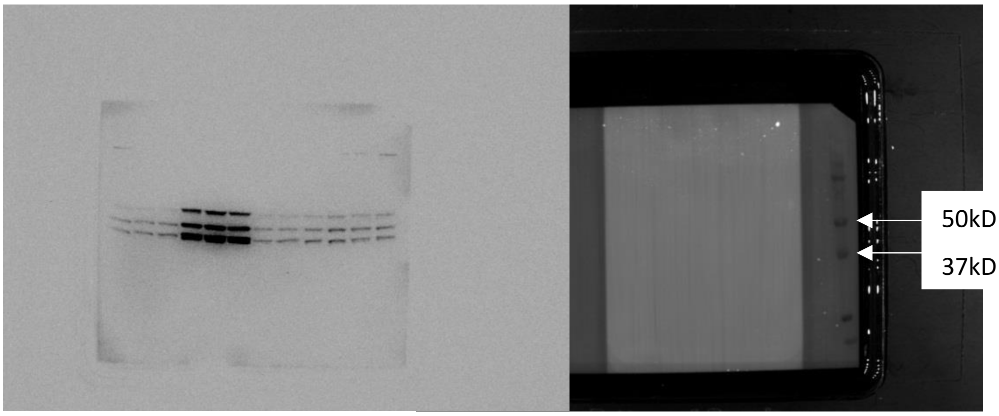

pJNK

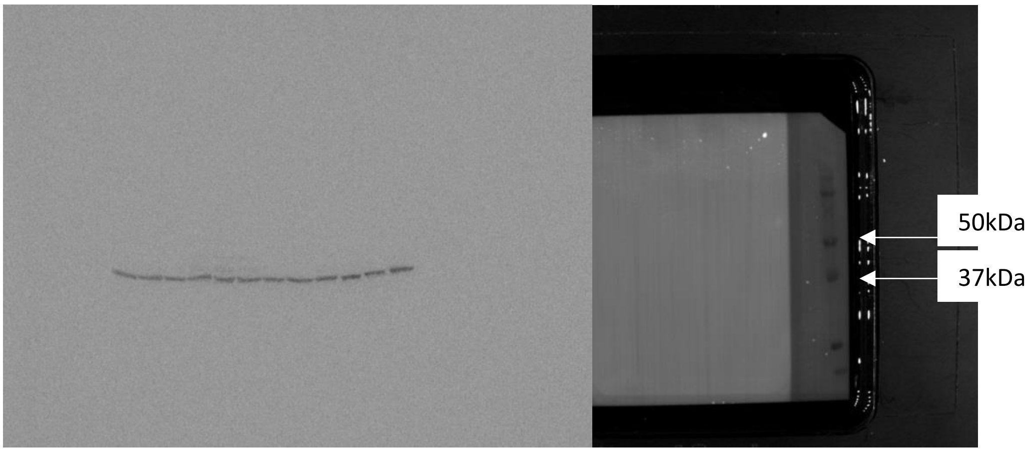

Gapdh

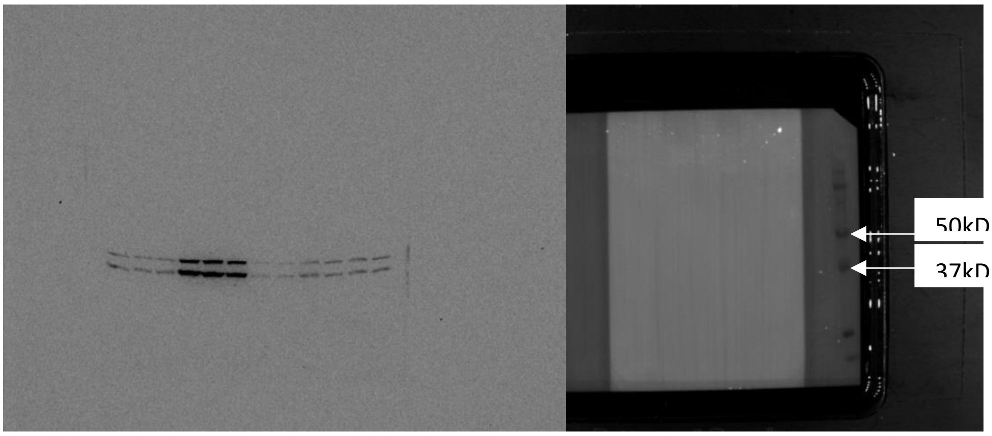

pERK

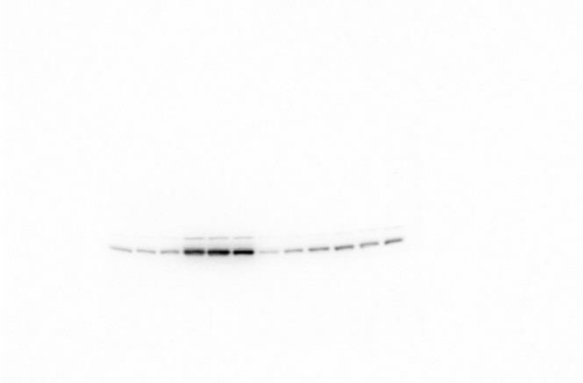

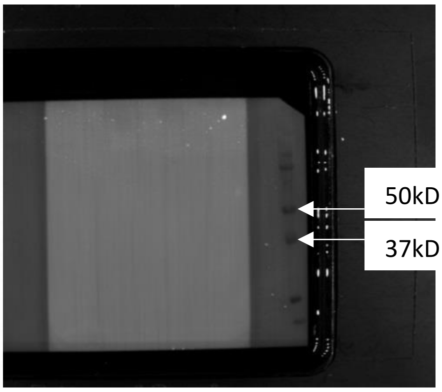

p-p38

Supplementary Table 1

Table 1. RT-qPCR primer details.

Gene	Accession	Forward primer	Reverse primer	Amplicon size (bp)	STD forward primer	STD reverse primer
Gapdh	NM_001289726	AAT GTG TCC GTC GTG GAT CT	AGA CAA CCT GGT CCT CAG TG	133	GCA TTG TGG AAG GGC TCA TG	GGC ATC GAA GGT GGA AGA GT
Henmt1	NM_025723	AGG CAT CTC GTG TGA AAA GGA	TCA GAA TCA GCG GCA ACT CA	60	TGA CCC AAA ACC GAC ACC TT	GCC ACC AAC CAC ACT ATT GC
Tlr3	NM_126166	CAG GCG TCC TTG GAC TTG AA	TAG CTT GCT GAA CTG CGT GA	363		
Tlr4	NM_021297	GCT TGA ATC CCT GCA TAG AGG TAG	TGT CAT CAG GGA CTT TGC TGA G	81		
Tlr7	NM_001290755	AGA AAG ATG TCC TTG GCT CCC	CCGTGTCCACATCGA AAACAC	207		
Tlr8	NM_133212	TCT GGT CCA GCT ATA GAG CAC A	AAT CCA TGA CTG AGG GGG CA	123		
Il-6	NM_031168.1	CGG CCT TCC CTA CTTCACAA	TCA TTT CCA CGA TTT CCC AGA GA	129	GGA GCC CAC CAA GAA CGA T	TGG TCC TTA GCC ACT CCT TCT
Tnf-a	NM_013693.3	CAC CAC CAT CAA GGA CTC AA	GAG GCA ACC TGA CCA CTC TC	96	TCT GTG AAG GGA ATG GGT GT	GGC TGG CTC TGT GAG GAA
Ccl2	NM_011333.3	CTC ACC TGC TGC TAC TCA TTC A	CCA TTC CTT CTT GGG GTC A	153	CAC CAG CAC CAG CCA ACT	GCA TCA CAG TCC GAG TCA CA
Ccl5	NM_013653.3	CTG CTG CTT TGC CTA CCT CT	ACA CAC TTG GCG GTT CCT T	124	CCC TCA CCA TCA TCC TCA CT	TCA GAA TCA AGA GGC CCT CTA TCC
Cxcl10	NM_021274.2	GCT CAA GTG GCT GGG ATG	GAG GAC AAG GAG GGT GTG G	111	CGA TGG ATG GAC AGC AGA GAG CCT	GAC AAG GAG GGT GTG GGA GC A

Supplementary Table 2

Table 2. Details of statistical analysis output, related to Figures 2-7.

For the Kolmogorov-Smirnof test, note that some evidence for deviation from normality was tolerated, as mixed model ANOVA is relatively robust against deviations from normality and lack of homogeneity of variance, whatever the sample size (Glass, G.V., Peckham, P.D. and Saunders, J.R., Review of Educational Research, 1972, 42, pp. 237-288; Blanca MJ, Alarcón R, Arnau J, Bono R, and Bendayan R, 2017, Psicothema. 2017, 29:552-557; Knief, U. and Forstmaier, W., 2021, Res Methods. 10.3758/s13428-021-01587-5.

**Figure (*gene* /protein)**	**Source**	**F and p values**	**ANOVA post-hoc comparisons**<br>**+ Fisher-Pitman permutation one-tailed tests (red)**	**Normality test p value**	**Grubb's & Levene's test p values**
2B (JNK)	Treatment	F(3,24)=13.54,<br>p<0.0001	Vehicle vs. resiquimod (p=0.0002)<br>Vehicle vs. poly I:C (p=0.9328)<br>Vehicle vs. LPS (p=0.7715)<br>LPS vs. resiquimod (p=0.0017)	Veh/pIC/LPS:<br>p>0.150<br>R848:<br>p>0.150	P=0.324<br>P=0.952
	Isoform	F(2,24)=8.602,<br>p=0.0015	54 kDa vs. 46 kDa (p=0.0032)<br>54 kDa vs. 43 kDa (p=0.0052)<br>46 kDa vs. 43 kDa (p=0.9793)		
	Treatment *Isoform	F(6,24)=1.735,<br>p=0.1560	Vehicle (54 kDa) vs. resiquimod (54 kDa) (p=0.0096) (p=0.042)		
2D (ERK)	Treatment	F(3,40)=18.19,<br>p<0.0001	Vehicle vs. resiquimod (p=0.000)<br>Vehicle vs. poly I:C (p=0.6068)<br>Vehicle vs. LPS (p=0.9827)<br>LPS vs. resiquimod (p=0.0000)	Veh/pIC/LPS:<br>P=0.083<br>R848:<br>P=0.054	P=1.000<br>P=0.037
	Isoform	F(1,40)=0.009870,<br>p=0.9214			
	Treatment *Isoform	F(3,40)=0.1271,<br>p=0.9435	Vehicle (44 kDa) vs. resiquimod (44 kDa) (p=0.0084) (p=0.047)<br>Vehicle (42 kDa) vs. resiquimod (42 kDa) (p=0.0056) (p=0.047)		
2E (p38)	Between groups	F(3,20)=37.00,<br>p=0.000	Vehicle vs. resiquimod (p=0.048)	Veh/pIC/LPS:<br>p>0.150<br>R848:<br>P=0.075	P=0.596<br>P=0.038
3A	Between groups	F(3,83)=75.59,<br>p=0.000	Vehicle vs. LPS (p=0.000)<br>Vehicle vs. poly I:C (p>0.999)<br>Vehicle vs. resiquimod (p=0.000)		P=0.892<br>P=0.001
3B-1	Between groups	F(3,38)=0.36,<br>p=0.779	Vehicle vs. vehicle+JNK-IN-8 (p=0.932)<br>Vehicle vs. vehicle+PD98059 (p=0.840)<br>Vehicle vs. vehicle+SB203580 (p=1.000)		P=0.000<br>P=0.023
3B-2	Between groups	F(3,39)=14.79,<br>p=0.000	LPS vs. LPS+JNK-IN-8 (p=0.004)<br>LPS vs. LPS+PD98059 (p=0.000)<br>LPS vs. LPS+SB203580 (p=0.724)		P=0.411<br>P=0.990
3B-3	Between groups	F(3,42)=8.18,<br>p=0.0000	Resiquimod vs. resiquimod+JNK-IN-8 (p=0.074)<br>Resiquimod vs. resiquimod+PD98059 (p=0.999)<br>Resiquimod vs. resiquimod+SB203580 (p=0.067)		P=0.892<br>P=0.601
4A (*Il-6*)	Treatment	F(3,35)=238.58,<br>p=0.000	Vehicle vs. LPS (p=0.000)<br>Vehicle vs. poly I:C (p=0.885)<br>Vehicle vs. resiquimod (p=0.000)<br>LPS vs. resiquimod (p=0.987)	Veh/pIC:<br>P>0.150<br>R848/LPS:<br>P=0.121	P=1.000<br>P=0.247

	Time	F(2,35)=82.68,<br>p=0.000	24hr vs. 8hr (p=0.000)<br>8hr vs. 0.5hr (p=0.000)<br>24hr vs. 0.5hr (p=0.000)		
	Treatment<br>*Time	F(6,35)=26.01,<br>p=0.000	Vehicle (0.5hr) vs. LPS (0.5hr) (p=0.006)<br>(p=0.033)<br>Vehicle (8hr) vs. LPS (8hr) (p=0.000)<br>(p=0.015)<br>Vehicle (24hr) vs. LPS (24hr) (p=0.000)<br>(p=0.014)<br>Vehicle (0.5hr) vs. resiquimod (0.5hr)<br>(p=0.000) (p=0.010)<br>Vehicle (8hr) vs. resiquimod (8hr)<br>(p=0.000) (p=0.017)<br>Vehicle (24hr) vs. resiquimod (24hr)<br>(p=0.000) (p=0.015)<br>LPS (0.5hr) vs. LPS (8hr) (p=0.000)<br>LPS (0.5hr) vs. LPS (24hr) (p=0.000)<br>Resiquimod (0.5hr) vs. resiquimod (8hr)<br>(p=0.000)<br>Resiquimod (0.5hr) vs. resiquimod (24hr)<br>(p=0.000)<br>LPS (0.5hr) vs. resiquimod (0.5hr)<br>(p=0.954)<br>LPS (8hr) vs. resiquimod (8hr) (p=1.000)<br>LPS (24hr) vs. resiquimod (24hr)<br>(p=1.000)		
4B (IL-6)	Treatment	F(3,24)=559.90,<br>p=0.000	Vehicle vs. LPS (p=0.000)<br>Vehicle vs. poly I:C (p=0.970)<br>Vehicle vs. resiquimod (p=0.000)<br>LPS vs. resiquimod (p=0.987)	Veh/pIC:<br>P>0.150<br>R848/LPS:<br>P=0.066	P=1.000<br>P=0.892
	Passage	F(2,24)=38.96,<br>p=0.000	1 vs. 2 (p=0.000)<br>1 vs. 3 (p=0.000<br>2 vs. 3 (p=0.052)		
	Time	F(1,24)=228.88,<br>p=0.000	24hr vs. 8hr (p=0.000)		
	Treatment<br>*Time	F(3,24)=108.74,<br>p=0.000	Vehicle (8hr) vs. LPS (8hr) (p=0.000)<br>Vehicle (24hr) vs. LPS (24hr) (p=0.000)<br>Vehicle (8hr) vs. resiquimod (8hr)<br>(p=0.000)<br>Vehicle (24hr) vs. resiquimod (24hr)<br>(p=0.000)<br>LPS (8hr) vs. LPS (24hr) (p=0.000)<br>Resiquimod (8hr) vs. resiquimod (24hr)<br>(p=0.000)<br>LPS (8hr) vs. resiquimod (8hr) (p=0.000)<br>LPS (24hr) vs. resiquimod (24hr)<br>(p=0.065)		
4C (*Tnf-α*)	Treatment	F(3,35)=76.18,<br>p=0.000	Vehicle vs. LPS (p=0.000)<br>Vehicle vs. poly I:C (p=0.982)<br>Vehicle vs. resiquimod (p=0.000)<br>LPS vs. resiquimod (p=0.774)	Veh/pIC:<br>P>0.150<br>R848/LPS:<br>P=0.047	P=1.000<br>P=0.365
	Time	F(2,35)=11.79,<br>p=0.000	24hr vs. 8hr (p=0.001)<br>8hr vs. 0.5hr (p=0.877)<br>24hr vs. 0.5hr (p=0.000)		
	Treatment<br>*Time	F(6,35)=4.27,<br>p=0.003	Vehicle (0.5hr) vs. LPS (0.5hr) (p=0.000)<br>(p=0.014)<br>Vehicle (8hr) vs. LPS (8hr) (p=0.000)<br>(p=0.018)		

			Vehicle (24hr) vs. LPS (24hr) (p=0.003) (p=0.013)<br>Vehicle (0.5hr) vs. resiquimod (0.5hr) (p=0.000) (p=0.010)<br>Vehicle (8hr) vs. resiquimod (8hr) (p=0.000) (p=0.014)<br>Vehicle (24hr) vs. resiquimod (24hr) (p=0.062) (p=0.016)<br>LPS (0.5hr) vs. LPS (8hr) (p=1.000)<br>LPS (0.5hr) vs. LPS (24hr) (p=0.352)<br>Resiquimod (0.5hr) vs. resiquimod (8hr) (p=0.673)<br>Resiquimod (0.5hr) vs. resiquimod (24hr) (p=0.000)<br>LPS (0.5hr) vs. resiquimod (0.5hr) (p=0.367)<br>LPS (8hr) vs. resiquimod (8hr) (p=1.000)<br>LPS (24hr) vs. resiquimod (24hr) (p=0.992)		
4D (TNF-α)	Treatment	F(3,44)=83.78, p=0.000	Vehicle vs. LPS (p=0.000)<br>Vehicle vs. poly I:C (p=0.909)<br>Vehicle vs. resiquimod (p=0.000)<br>LPS vs. resiquimod (p=0.000)	Veh/pIC: P>0.150<br>R848/LPS: P=0.044	P=1.000<br>P=0.005
	Passage	F(2,44)=49.65, p=0.000	1 vs. 2 (p=0.000)<br>1 vs. 3 (p=0.000)<br>2 vs. 3 (p=0.012)		
	Time	F(2,44)=30.59, p=0.000	24hr vs. 8hr (p=0.001)<br>8hr vs. 0.5hr (p=0.000)<br>24hr vs. 0.5hr (p=0.000)		
	Treatment *Time	F(6,44)=11.85, p=0.000	LPS (0.5hr) vs. resiquimod (0.5hr) (p=0.158)<br>Vehicle (8hr) vs. LPS (8hr) (p=0.003)<br>Vehicle (24hr) vs. LPS (24hr) (p=0.000)<br>Vehicle (0.5hr) vs. resiquimod (0.5hr) (p=0.009)<br>Vehicle (8hr) vs. resiquimod (8hr) (p=0.000)<br>Vehicle (24hr) vs. resiquimod (24hr) (p=0.000)<br>LPS (0.5hr) vs. LPS (8hr) (p=0.104)<br>LPS (0.5hr) vs. LPS (24hr) (p=0.000)<br>Resiquimod (0.5hr) vs. resiquimod (8hr) (p=0.000)<br>Resiquimod (0.5hr) vs. resiquimod (24hr) (p=0.000)<br>LPS (0.5hr) vs. resiquimod (0.5hr) (p=0.158)<br>LPS (8hr) vs. resiquimod (8hr) (p=0.000)<br>LPS (24hr) vs. resiquimod (24hr) (p=0.994)		
5A (*Ccl2*)	Treatment	F(3,35)=112.39, p=0.000	Vehicle vs. LPS (p=0.000)<br>Vehicle vs. poly I:C (p=1.000)<br>Vehicle vs. resiquimod (p=0.000)<br>LPS vs. resiquimod (p=0.796)	Veh/pIC: P=0.040<br>R848/LPS: P<0.010	P=1.000<br>P=0.957
	Time	F(2,35)=51.59, p=0.000	24hr vs. 8hr (p=0.573)<br>8hr vs. 0.5hr (p=0.000)<br>24hr vs. 0.5hr (p=0.000)		

	Treatment *Time	F(6,35)=16.85, p=0.000	Vehicle (0.5hr) vs. LPS (0.5hr) (p=0.960) p=0.071)<br>Vehicle (8hr) vs. LPS (8hr) (p=0.000) (p=0.010)<br>Vehicle (24hr) vs. LPS (24hr) (p=0.000) (p=0.017)<br>Vehicle (0.5hr) vs. resiquimod (0.5hr) (p=0.345) (p=0.028)<br>Vehicle (8hr) vs. resiquimod (8hr) (p=0.000) (p=0.020)<br>Vehicle (24hr) vs. resiquimod (24hr) (p=0.000) (p=0.014)<br>LPS (0.5hr) vs. LPS (8hr) (p=0.000)<br>LPS (0.5hr) vs. LPS (24hr) (p=0.000)<br>Resiquimod (0.5hr) vs. resiquimod (8hr) (p=0.000)<br>Resiquimod (0.5hr) vs. resiquimod (24hr) (p=0.000)<br>LPS (0.5hr) vs. resiquimod (0.5hr) (p=0.988)<br>LPS (8hr) vs. resiquimod (8hr) (p=1.000)<br>LPS (24hr) vs. resiquimod (24hr) (p=1.000)		
5B (CCL2)	Treatment	F(3,33)=25.62, p=0.000	Vehicle vs. LPS (p=0.031)<br>Vehicle vs. poly I:C (p=0.862)<br>Vehicle vs. resiquimod (p=0.000)<br>LPS vs. resiquimod (p=0.001)	Veh/pIC: P>0.150<br>R848/LPS: P>0.150	P=0.079<br>P=0.296
	Passage	F(2,33)=48.79, p=0.000	1 vs. 2 (p=0.000)<br>1 vs. 3 (p=0.605)<br>2 vs. 3 (p=0.000)		
	Time	F(2,33)=55.17, p=0.000	24hr vs. 8hr (p=0.000)<br>8hr vs. 0.5hr (p=0.988)<br>24hr vs. 0.5hr (p=0.000)		
	Treatment *Time	F(6,33)=2.17, p=0.072	Vehicle (0.5hr) vs. LPS (0.5hr) (p=1.000)<br>Vehicle (8hr) vs. LPS (8hr) (p=0.998)<br>Vehicle (24hr) vs. LPS (24hr) (p=0.000)<br>Vehicle (0.5hr) vs. resiquimod (0.5hr) (p=0.975)<br>Vehicle (8hr) vs. resiquimod (8hr) (p=0.990)<br>Vehicle (24hr) vs. resiquimod (24hr) (p=0.000)<br>LPS (0.5hr) vs. LPS (8hr) (p=1.000)<br>LPS (0.5hr) vs. LPS (24hr) (p=0.000)<br>Resiquimod (0.5hr) vs. resiquimod (8hr) (p=1.000)<br>Resiquimod (0.5hr) vs. resiquimod (24hr) (p=0.000)<br>LPS (0.5hr) vs. resiquimod (0.5hr) (p=0.992)<br>LPS (8hr) vs. resiquimod (8hr) (p=0.690)<br>LPS (24hr) vs. resiquimod (24hr) (p=0.010)		
5C (*Ccl5*)	Treatment	F(3,36)=80.20, p=0.000	Vehicle vs. LPS (p=0.000)<br>Vehicle vs. poly I:C (p=0.497)<br>Vehicle vs. resiquimod (p=0.000)<br>LPS vs. resiquimod (p=0.000)	Veh/pIC: P=0.026<br>R848/LPS: P>0.150	P=0.0164<br>P=0.032

	Time	F(2,36)=60.30,<br>p=0.000	24hr vs. 8hr (p=0.155)<br>8hr vs. 0.5hr (p=0.000)<br>24hr vs. 0.5hr (p=0.000)		
	Treatment<br>*Time	F(6,36)=23.67,<br>p=0.000	Vehicle (0.5hr) vs. LPS (0.5hr) (p=1.000) (p=0.315)<br>Vehicle (8hr) vs. LPS (8hr) (p=0.000) (p=0.016)<br>Vehicle (24hr) vs. LPS (24hr) (p=0.000) (p=0.019)<br>Vehicle (0.5hr) vs. resiquimod (0.5hr) (p=1.000) (p=0.375)<br>Vehicle (8hr) vs. resiquimod (8hr) (p=0.000) (p=0.019)<br>Vehicle (24hr) vs. resiquimod (24hr) (p=0.000) (p=0.017)<br>LPS (0.5hr) vs. LPS (8hr) (p=0.000)<br>LPS (0.5hr) vs. LPS (24hr) (p=0.000)<br>Resiquimod (0.5hr) vs. resiquimod (8hr) (p=0.000)<br>Resiquimod (0.5hr) vs. resiquimod (24hr) (p=0.000)<br>LPS (0.5hr) vs. resiquimod (0.5hr) (p=1.000)<br>LPS (8hr) vs. resiquimod (8hr) (p=0.000)<br>LPS (24hr) vs. resiquimod (24hr) (p=0.006)		
5D<br>(CCL5)	Treatment	F(3,33)=4.01,<br>p=0.015	Vehicle vs. LPS (p=0.737)<br>Vehicle vs. poly I:C (p=0.902)<br>Vehicle vs. resiquimod (p=0.214)<br>LPS vs. resiquimod (p=0.016)	Veh/pIC:<br>P>0.150<br>R848/LPS:<br>P>0.150	P=1.000<br>P=0.667
	Passage	F(2,33)=9.04,<br>p=0.001	1 vs. 2 (p=0.334)<br>1 vs. 3 (p=0.124)<br>2 vs. 3 (p=0.785)		
	Time	F(2,33)=4.71,<br>p=0.016	24hr vs. 8hr (p=0.012)<br>8hr vs. 0.5hr (p=0.465)<br>24hr vs. 0.5hr (p=0.145)		
	Treatment<br>*Time	F(6,33)=2.17,<br>p=0.071	Vehicle (0.5hr) vs. LPS (0.5hr) (p=1.000)<br>Vehicle (8hr) vs. LPS (8hr) (p=0.893)<br>Vehicle (24hr) vs. LPS (24hr) (p=1.000)<br>Vehicle (0.5hr) vs. resiquimod (0.5hr) (p=0.999)<br>Vehicle (8hr) vs. resiquimod (8hr) (p=1.000)<br>Vehicle (24hr) vs. resiquimod (24hr) (p=0.110)<br>LPS (0.5hr) vs. LPS (8hr) (p=0.999)<br>LPS (0.5hr) vs. LPS (24hr) (p=0.999)<br>Resiquimod (0.5hr) vs. resiquimod (8hr) (p=1.000)<br>Resiquimod (0.5hr) vs. resiquimod (24hr) (p=0.078)<br>LPS (0.5hr) vs. resiquimod (0.5hr) (p=0.997)<br>LPS (8hr) vs. resiquimod (8hr) (p=0.994)<br>LPS (24hr) vs. resiquimod (24hr) (p=0.058)		
5E<br>(*Cxcl10*)	Treatment	F(3,36)=45.24,<br>p=0.000	Vehicle vs. LPS (p=0.000)<br>Vehicle vs. poly I:C (p=0.821)	Veh/pIC:<br>P>0.150	P=0.133<br>P=0.681

			Vehicle vs. resiquimod (p=0.000)<br>LPS vs. resiquimod (p=0.000)	R848/LPS:<br>P<0.010	
	Time	F(2,36)=8.49,<br>p=0.001	24hr vs. 8hr (p=0.060)<br>8hr vs. 0.5hr (p=0.001)<br>24hr vs. 0.5hr (p=0.202)		
	Treatment *Time	F(6,36)=7.61,<br>p=0.000	Vehicle (0.5hr) vs. LPS (0.5hr) (p=0.885) (p=0.015)<br>Vehicle (8hr) vs. LPS (8hr) (p=0.000) (p=0.014)<br>Vehicle (24hr) vs. LPS (24hr) (p=0.000) (p=0.015)<br>Vehicle (0.5hr) vs. resiquimod (0.5hr) (p=0.056) (p=0.015)<br>Vehicle (8hr) vs. resiquimod (8hr) (p=0.030) (p=0.011)<br>Vehicle (24hr) vs. resiquimod (24hr) (p=0.562) (p=0.062)<br>LPS (0.5hr) vs. LPS (8hr) (p=0.000)<br>LPS (0.5hr) vs. LPS (24hr) (p=0.001)<br>Resiquimod (0.5hr) vs. resiquimod (8hr) (p=1.000)<br>Resiquimod (0.5hr) vs. resiquimod (24hr) (p=0.936)<br>LPS (0.5hr) vs. resiquimod (0.5hr) (p=0.802)<br>LPS (8hr) vs. resiquimod (8hr) (p=0.000)<br>LPS (24hr) vs. resiquimod (24hr) (p=0.001)		
5F (CXCL10)	Treatment	F(3,33)=1.29,<br>p=0.294	Vehicle vs. LPS (p=0.707)<br>Vehicle vs. poly I:C (p=0.948)<br>Vehicle vs. resiquimod (p=0.851)<br>LPS vs. resiquimod (p=0.256)	Veh/pIC:<br>P=0.132<br>R848/LPS:<br>P<0.010	P=0.527<br>P=0.543
	Passage	F(2,33)=9.04,<br>p=0.001	1 vs. 2 (p=0.027)<br>1 vs. 3 (p=0.001)<br>2 vs. 3 (p=0.259)		
	Time	F(2,33)=1.65,<br>p=0.207	24hr vs. 8hr (p=0.179)<br>8hr vs. 0.5hr (p=0.641)<br>24hr vs. 0.5hr (p=0.635)		
	Treatment *Time	F(6,33)=0.88,<br>p=0.521	Vehicle (0.5hr) vs. LPS (0.5hr) (p=1.000)<br>Vehicle (8hr) vs. LPS (8hr) (p=0.965)<br>Vehicle (24hr) vs. LPS (24hr) (p=1.000)<br>Vehicle (0.5hr) vs. resiquimod (0.5hr) (p=1.000)<br>Vehicle (8hr) vs. resiquimod (8hr) (p=1.000)<br>Vehicle (24hr) vs. resiquimod (24hr) (p=0.952)<br>LPS (0.5hr) vs. LPS (8hr) (p=0.999)<br>LPS (0.5hr) vs. LPS (24hr) (p=1.000)<br>Resiquimod (0.5hr) vs. resiquimod (8hr) (p=0.999)<br>Resiquimod (0.5hr) vs. resiquimod (24hr) (p=0.953)<br>LPS (0.5hr) vs. resiquimod (0.5hr) (p=0.998)<br>LPS (8hr) vs. resiquimod (8hr) (p=1.000)<br>LPS (24hr) vs. resiquimod (24hr) (p=0.898)		

6A-C (*Il-6*)	Treatment	F(2,66)=1116.36, p=0.000	Vehicle vs. LPS (p=0.000)<br>Vehicle vs. resiquimod (p=0.000)<br>LPS vs. resiquimod (p=0.000)	Veh: P<0.010<br>R848/LPS: P>0.150	P=1.000<br>P=0.000
	Inhibitor	F(3,66)=5.14, p=0.003	Control vs. JNK-IN-8 (p=0.790)<br>Control vs. PD98059 (p=0.003)<br>Control vs. SB203580 (p=0.022)		
	Passage	F(2,66)=3.57, p=0.034			
	Treatment *Inhibitor	F(6,66)=9.31, p=0.000	Vehicle, none vs. vehicle, JNK-IN-8 (p=0.714)<br>Vehicle, none vs vehicle, PD98059 (p=0.723)<br>Vehicle, none vs. vehicle, SB203580 (p=0.999)<br>LPS, none vs. LPS, JNK-IN-8 (p=0.749)<br>LPS, none vs. LPS, PD98059 (p=1.000)<br>LPS none, vs. LPS, SB203580 (p=0.968)<br>Resiquimod, none, vs. resiquimod, JNK-IN-8 (p=0.413)<br>Resiquimod, none vs. resiquimod, PD98059 (p=0.002)<br>Resiquimod, none vs. resiquimod, SB203580 (p=0.012)<br>Vehicle, none vs. LPS, none (p=0.000)<br>Vehicle, JNK-IN-8 vs. LPS, JNK-IN-8 (p=0.000)<br>Vehicle, PD98059 vs. LPS, PD98059 (p=0.000)<br>Vehicle, SB203580 vs. LPS, SB203580 (p=0.000)<br>Vehicle, none vs. resiquimod, none (p=0.000)<br>Vehicle, JNK-IN-8 vs. resiquimod, JNK-IN-8 (p=0.000)<br>Vehicle, PD98059 vs. resiquimod, PD98059 (p=0.000)<br>Vehicle, SB203580 vs. resiquimod, SB203580 (p=0.000)<br>LPS, none vs. resiquimod, none (p=0.002)<br>LPS, JNK-IN-8 vs. resiquimod, JNK-IN-8<br>LPS, PD98059 vs. resiquimod, PD98059 (p=0.000)<br>LPS, SB203580 vs. resiquimod, SB203580 (p=0.000)		
6D-F (*Tnf-α*)	Treatment	F(2,66)=350.46, p=0.000	Vehicle vs. LPS (p=0.000)<br>Vehicle vs. resiquimod (p=0.000)<br>LPS vs. resiquimod (p=0.000)	Veh: P>0.150<br>R848/LPS: P>0.150	P=0.892<br>P=0.000
	Inhibitor	F(3,66)=17.36, p=0.000	Control vs. JNK-IN-8 (p=0.760)<br>Control vs. PD98059 (p=0.002)<br>Control vs. SB203580 (p=0.007)		
	Passage	F(2,66)=0.11, p=0.894			

	Treatment *Inhibitor	F(6,66)=2.41, p=0.036	Vehicle, none vs. vehicle, JNK-IN-8 (p=1.000)<br>Vehicle, none vs vehicle, PD98059 (p=0.000)<br>Vehicle, none vs. vehicle, SB203580 (p=1.000)<br>LPS, none vs. LPS, JNK-IN-8 (p=1.000)<br>LPS, none vs. LPS, PD98059 (p=0.639)<br>LPS none, vs. LPS, SB203580 (p=0.125)<br>Resiquimod, none, vs. resiquimod, JNK-IN-8 (p=0.875)<br>Resiquimod, none vs. resiquimod, PD98059 (p=1.000)<br>Resiquimod, none vs. resiquimod, SB203580 (p=0.825)<br>Vehicle, none vs. LPS, none (p=0.000)<br>Vehicle, JNK-IN-8 vs. LPS, JNK-IN-8 (p=0.000)<br>Vehicle, PD98059 vs. LPS, PD98059 (p=0.000)<br>Vehicle, SB203580 vs. LPS, SB203580 (p=0.000)<br>Vehicle, none vs. resiquimod, none (p=0.000)<br>Vehicle, JNK-IN-8 vs. resiquimod, JNK-IN-8 (p=0.000)<br>Vehicle, PD98059 vs. resiquimod, PD98059 (p=0.000)<br>Vehicle, SB203580 vs. resiquimod, SB203580 (p=0.000)<br>LPS, none vs. resiquimod, none (p=0.041)<br>LPS, JNK-IN-8 vs. resiquimod, JNK-IN-8 (p=0.968)<br>LPS, PD98059 vs. resiquimod, PD98059 (p=0.003)<br>LPS, SB203580 vs. resiquimod, SB203580 (p=0.767)		
6G-I (TNF-α)	Treatment	F(2,35)=86.72, p=0.000	Vehicle vs. LPS (p=1.000)<br>Vehicle vs. resiquimod (p=0.000)<br>LPS vs. resiquimod (p=0.000)	Veh: P>0.150<br>R848/LPS: P=0.037	P=0.272<br>P=0.790
	Inhibitor	F(3,35)=7.97. p=0.003	Control vs. JNK-IN-8 (p=0.002)<br>Control vs. PD98059 (p=0.611)<br>Control vs. SB203580 (p=0.970)		
	Passage	F(2,35)=12.18. p=0.003	1 vs. 2 (p=0.001)<br>1 vs. 3 (p=0.000)<br>2 vs. 3 (p=0.705)		
	Treatment *Inhibitor	F(6,35)=5.65, p=0.000	Vehicle, none vs. vehicle, JNK-IN-8 (p=0.972)<br>Vehicle, none vs vehicle, PD98059 (p=1.000)<br>Vehicle, none vs. vehicle, SB203580 (p=0.995)<br>LPS, none vs. LPS, JNK-IN-8 (p=1.000)<br>LPS, none vs. LPS, PD98059 (p=1.000)<br>LPS none, vs. LPS, SB203580 (p=0.620)		

			Resiquimod, none, vs. resiquimod, JNK-IN-8 (p=0.000)<br>Resiquimod, none vs. resiquimod, PD98059 (p=0.842)<br>Resiquimod, none vs. resiquimod, SB203580 (p=1.000)<br>Vehicle, none vs. LPS, none (p=0.956)<br>Vehicle, JNK-IN-8 vs. LPS, JNK-IN-8 (p=1.000)<br>Vehicle, PD98059 vs. LPS, PD98059 (p=0.999)<br>Vehicle, SB203580 vs. LPS, SB203580 (p=0.839)<br>Vehicle, none vs. resiquimod, none (p=0.000)<br>Vehicle, JNK-IN-8 vs. resiquimod, JNK-IN-8 (p=0.565)<br>Vehicle, PD98059 vs. resiquimod, PD98059 (p=0.000)<br>Vehicle, SB203580 vs. resiquimod, SB203580 (p=0.000)<br>LPS, none vs. resiquimod, none (p=0.000)<br>LPS, JNK-IN-8 vs. resiquimod, JNK-IN-8 (p=0.889)<br>LPS, PD98059 vs. resiquimod, PD98059 (p=0.000)<br>LPS, SB203580 vs. resiquimod, SB203580 (p=0.000)		
7A-C (*Ccl2*)	Treatment	F(2,66)=360.50, p=0.000	Vehicle vs. LPS (p=0.000)<br>Vehicle vs. resiquimod (p=0.000)<br>LPS vs. resiquimod (p=0.000)	Veh: P>0.150<br>R848/LPS: P>0.150	P=1.000<br>P=0.017
	Inhibitor	F(3,66)=20.84, p=0.000	Control vs. JNK-IN-8 (p=0.998)<br>Control vs. PD98059 (p=0.023)<br>Control vs. SB203580 (p=0.000)		
	Passage	F(2,66)=1.83, p=0.168			
	Treatment *Inhibitor	F(6,66)=2.85, p=0.016	Vehicle, none vs. vehicle, JNK-IN-8 (p=0.998)<br>Vehicle, none vs vehicle, PD98059 (p=0.031)<br>Vehicle, none vs. vehicle, SB203580 (p=0.999)<br>LPS, none vs. LPS, JNK-IN-8 (p=0.976)<br>LPS, none vs. LPS, PD98059 (p=0.800)<br>LPS none, vs. LPS, SB203580 (p=0.001)<br>Resiquimod, none, vs. resiquimod, JNK-IN-8 (p=0.999)<br>Resiquimod, none vs. resiquimod, PD98059 (p=1.000)<br>Resiquimod, none vs. resiquimod, SB203580 (p=0.340)<br>Vehicle, none vs. LPS, none (p=0.000)<br>Vehicle, JNK-IN-8 vs. LPS, JNK-IN-8 (p=0.000)<br>Vehicle, PD98059 vs. LPS, PD98059 (p=0.000)		

			Vehicle, SB203580 vs. LPS, SB203580 (p=0.000)<br>Vehicle, none vs. resiquimod, none (p=0.000)<br>Vehicle, JNK-IN-8 vs. resiquimod, JNK-IN-8 (p=0.000)<br>Vehicle, PD98059 vs. resiquimod, PD98059 (p=0.000)<br>Vehicle, SB203580 vs. resiquimod, SB203580 (p=0.000)<br>LPS, none vs. resiquimod, none (p=0.010)<br>LPS, JNK-IN-8 vs. resiquimod, JNK-IN-8 (p=0.953)<br>LPS, PD98059 vs. resiquimod, PD98059 (p=0.001)<br>LPS, SB203580 vs. resiquimod, SB203580 (p=0.891)		
7D-F (CCL2)	Treatment	F(2,45)=7.22, p=0.002	Vehicle vs. LPS (p=0.096)<br>Vehicle vs. resiquimod (p=0.313)<br>Resiquimod vs. LPS (p=0.001)	Veh: P>0.150<br>R848/LPS: P=0.021	P=0.005<br>P=0.411
	Inhibitor	F(3,45)=6.97, p=0.001	Control vs. JNK-IN-8 (p=0.003)<br>Control vs. PD98059 (p=0.999)<br>Control vs. SB203580 (p=0.700)		
	Passage	F(2,45)=6.73, p=0.003	1 vs. 2 (p=0.632)<br>1 vs. 3 (p=0.003)<br>2 vs. 3 (p=0.028)		
	Treatment *Inhibitor	F(6,45)=1.69, p=0.147	Vehicle, none vs. vehicle, JNK-IN-8 (p=0.997)<br>Vehicle, none vs vehicle, PD98059 (p=1.000)<br>Vehicle, none vs. vehicle, SB203580 (p=0.998)<br>LPS, none vs. LPS, JNK-IN-8 (p=0.006)<br>LPS, none vs. LPS, PD98059 (p=1.000)<br>LPS none, vs. LPS, SB203580 (p=0.888)<br>Resiquimod, none, vs. resiquimod, JNK-IN-8 (p=0.935)<br>Resiquimod, none vs. resiquimod, PD98059 (p=1.000)<br>Resiquimod, none vs. resiquimod, SB203580 (p=0.902)<br>Vehicle, none vs. LPS, none (p=0.463)<br>Vehicle, JNK-IN-8 vs. LPS, JNK-IN-8 (p=1.000)<br>Vehicle, PD98059 vs. LPS, PD98059 (p=0.842)<br>Vehicle, SB203580 vs. LPS, SB203580 (p=1.000)<br>Vehicle, none vs. resiquimod, none (p=1.000)<br>Vehicle, JNK-IN-8 vs. resiquimod, JNK-IN-8 (p=1.000)<br>Vehicle, PD98059 vs. resiquimod, PD98059 (p=0.995)<br>Vehicle, SB203580 vs. resiquimod, SB203580 (p=0.401)		

			LPS, none vs. resiquimod, none (p=0.354)<br>LPS, JNK-IN-8 vs. resiquimod, JNK-IN-8 (p=1.000)<br>LPS, PD98059 vs. resiquimod, PD98059 (p=0.257)<br>LPS, SB203580 vs. resiquimod, SB203580 (p=0.376)		
7G-I (*Ccl5*)	Treatment	F(2,66)=331.79, p=0.000	Vehicle vs. LPS (p=0.000)<br>Vehicle vs. resiquimod (p=0.000)<br>LPS vs. resiquimod (p=0.005)	Veh:<br>P=0.038<br>R848/LPS:<br>P>0.150	P=0.584<br>P=0.014
	Inhibitor	F(3,66)=15.06. p=0.000	Control vs. JNK-IN-8 (p=0.715)<br>Control vs. PD98059 (p=0.674)<br>Control vs. SB203580 (p=0.000)		
	Passage	F(2,66)=4.13, p=0.020			
	Treatment *Inhibitor	F(6,66)=6.62, p=0.000	Vehicle, none vs. vehicle, JNK-IN-8 (p=1.000)<br>Vehicle, none vs vehicle, PD98059 (p=0.948)<br>Vehicle, none vs. vehicle, SB203580 (p=1.000)<br>LPS, none vs. LPS, JNK-IN-8 (p=0.946)<br>LPS, none vs. LPS, PD98059 (p=1.000)<br>LPS none, vs. LPS, SB203580 (p=0.056)<br>Resiquimod, none, vs. resiquimod, JNK-IN-8 (p=1.000)<br>Resiquimod, none vs. resiquimod, PD98059 (p=0.137)<br>Resiquimod, none vs. resiquimod, SB203580 (p=0.000)<br>Vehicle, none vs. LPS, none (p=0.000)<br>Vehicle, JNK-IN-8 vs. LPS, JNK-IN-8 (p=0.000)<br>Vehicle, PD98059 vs. LPS, PD98059 (p=0.000)<br>Vehicle, SB203580 vs. LPS, SB203580 (p=0.000)<br>Vehicle, none vs. resiquimod, none (p=0.000)<br>Vehicle, JNK-IN-8 vs. resiquimod, JNK-IN-8 (0.000)<br>Vehicle, PD98059 vs. resiquimod, PD98059 (p=0.000)<br>Vehicle, SB203580 vs. resiquimod, SB203580 (p=0.000)<br>LPS, none vs. resiquimod, none (p=0.054)<br>LPS, JNK-IN-8 vs. resiquimod, JNK-IN-8 (p=0.005)<br>LPS, PD98059 vs. resiquimod, PD98059 (p=1.000)<br>LPS, SB203580 vs. resiquimod, SB203580 (p=1.000)		
7J-L (CCL5)	Treatment	F(2,41)=2.85, p=0.069	Vehicle vs. LPS (p=0.168)<br>Vehicle vs. resiquimod (p=0.092)<br>LPS vs. resiquimod (p=0.941)	Veh:<br>P>0.150<br>R848/LPS:	P=0.679<br>P=0.764

				P=0.141	
	Inhibitor	F(3,41)=4.65, p=0.008	Control vs. JNK-IN-8 (p=0.086)<br>Control vs. PD98059 (p=0.908)<br>Control vs. SB203580 (p=0.820)		
	Passage	F(2,41)=3.32, p=0.046	1 vs. 2 (p=0.095)<br>1 vs. 3 (p=0.053)<br>2 vs. 3 (p=0.959)		
	Treatment *Inhibitor	F(6,41)=3.00, p=0.016	Vehicle, none vs. vehicle, JNK-IN-8 (p=0.999)<br>Vehicle, none vs vehicle, PD98059 (p=1.000)<br>Vehicle, none vs. vehicle, SB203580 (p=0.139)<br>LPS, none vs. LPS, JNK-IN-8 (p=0.065)<br>LPS, none vs. LPS, PD98059 (p=1.000)<br>LPS none, vs. LPS, SB203580 (p=0.996)<br>Resiquimod, none, vs. resiquimod, JNK-IN-8 (p=1.000)<br>Resiquimod, none vs. resiquimod, PD98059 (p=0.958)<br>Resiquimod, none vs. resiquimod, SB203580 (p=1.000)<br>Vehicle, none vs. LPS, none (p=0.999)<br>Vehicle, JNK-IN-8 vs. LPS, JNK-IN-8 (p=0.671)<br>Vehicle, PD98059 vs. LPS, PD98059 (p=1.000)<br>Vehicle, SB203580 vs. LPS, SB203580 (p=0.119)<br>Vehicle, none vs. resiquimod, none (p=0.999)<br>Vehicle, JNK-IN-8 vs. resiquimod, JNK-IN-8 (p=1.000)<br>Vehicle, PD98059 vs. resiquimod, PD98059 (p=1.000)<br>Vehicle, SB203580 vs. resiquimod, SB203580 (p=0.009)<br>LPS, none vs. resiquimod, none (p=0.896)<br>LPS, JNK-IN-8 vs. resiquimod, JNK-IN-8 (p=0.936)<br>LPS, PD98059 vs. resiquimod, PD98059 (p=1.000)<br>LPS, SB203580 vs. resiquimod, SB203580 (p=1.000)		
7M-O (*Cxcl10*)	Treatment	F(2,66)=333.93, p=0.000	Vehicle vs. LPS (p=0.000)<br>Vehicle vs. resiquimod (p=0.000)<br>LPS vs. resiquimod (p=0.000)	Veh: P>0.150<br>R848/LPS: P=0.131	P=1.000<br>P=0.328
	Inhibitor	F(3,66)=4.91, p=0.004	Control vs. JNK-IN-8 (p=0.994)<br>Control vs. PD98059 (p=0.159)<br>Control vs. SB203580 (p=0.322)		
	Passage	F(2,66)=0.92, p=0.404			
	Treatment *Inhibitor	F(6,66)=1,21, p=0.312	Vehicle, none vs. vehicle, JNK-IN-8 (p=0.998)<br>Vehicle, none vs vehicle, PD98059 (p=0.339)		

			Vehicle, none vs. vehicle, SB203580 (p=1.000)<br>LPS, none vs. LPS, JNK-IN-8 (p=0.605)<br>LPS, none vs. LPS, PD98059 (p=1.000)<br>LPS none, vs. LPS, SB203580 (p=0.777)<br>Resiquimod, none, vs. resiquimod, JNK-IN-8 (p=0.996)<br>Resiquimod, none vs. resiquimod, PD98059 (p=0.994)<br>Resiquimod, none vs. resiquimod, SB203580 (p=0.999)<br>Vehicle, none vs. LPS, none (p=0.000)<br>Vehicle, JNK-IN-8 vs. LPS, JNK-IN-8 (p=0.000)<br>Vehicle, PD98059 vs. LPS, PD98059 (p=0.000)<br>Vehicle, SB203580 vs. LPS, SB203580 (p=0.000)<br>Vehicle, none vs. resiquimod, none (p=0.000)<br>Vehicle, JNK-IN-8 vs. resiquimod, JNK-IN-8 (p=0.000)<br>Vehicle, PD98059 vs. resiquimod, PD98059 (p=0.000)<br>Vehicle, SB203580 vs. resiquimod, SB203580 (p=0.000)<br>LPS, none vs. resiquimod, none (p=0.008)<br>LPS, JNK-IN-8 vs. resiquimod, JNK-IN-8 (p=0.000)<br>LPS, PD98059 vs. resiquimod, PD98059 (p=0.015)<br>LPS, SB203580 vs. resiquimod, SB203580 (p=0.002)		
